# Emerging, personalized, and minimally invasive technologies for periodontal and peri‐implant soft and hard tissue regenerative therapy

**DOI:** 10.1002/jper.70108

**Published:** 2026-09-14

**Authors:** Shayan Barootchi, Lorenzo Tavelli, Hamoun Sabri, William V. Giannobile, Darnell Kaigler

**Affiliations:** ^1^ Division of Periodontology Department of Oral Medicine Infection, and Immunity, Harvard School of Dental Medicine Boston Massachusetts USA; ^2^ Department of Periodontics & Oral Medicine University of Michigan School of Dentistry Ann Arbor Michigan USA; ^3^ Center for Clinical Research and Evidence Synthesis in Oral Tissue Regeneration (CRITERION) Ann Arbor Michigan USA

**Keywords:** augmentation, biologics, evidence‐based dentistry, network meta‐analysis, tissue engineering

## Abstract

**Background:**

Regenerative therapies have become central to periodontics and implant dentistry and are becoming more widely applied to reconstruct supporting bone and associated soft tissues. With increased recognition of the benefits of using biomimetic materials as scaffolds in contemporary regenerative technologies, the field is seeking to develop and optimize predictable, personalized regenerative strategies for hard and soft tissue reconstruction. This review aims to: (1) evaluate the current evidence on these approaches in the context of hard and soft tissue deficiencies around teeth and implants, (2) assess the clinical utility of these approaches, and (3) propose a framework for integrating these regenerative technologies into daily clinical practice.

**Methods:**

Applicable research on the treatment of hard and /or soft tissue deficiencies around teeth and dental implants were conducted. For soft tissue regeneration, data from eligible randomized clinical trials (RCTs) reporting the outcomes of gingival (for teeth, as gingival thickness [GT]) and mucosal thickness (implants, MT) with a bilaminar approach were analyzed using a mixed models network meta‐analysis. A meta‐analysis could not be performed to evaluate personalized, emerging technologies for bone regeneration given the lack of comparable clinical studies and as such, an alternative evaluative approach was undertaken and involved a qualitative assessment of the relevant literature highlighting innovations in these emerging modalities.

**Results:**

A total of 78 RCTs were included for a quantitative analysis on soft tissue regeneration. Fifty‐four reported the outcomes of GT with a bilaminar approach (a total of 2952 teeth), and 24 assessed MT around implants (851 implants). The analysis of GT for natural dentition showed that bilaminar approaches with any of the explored soft tissue grafts (connective tissue graft (CTG), human acellular dermal matrix (hADM), first generation collagen matrix (CMX), “volume stable” collagen matrix (VCMX) yield a statistically significant increase in GT (compared with untreated sites), with the highest estimate for CTG, followed by VCMX, hADM, pADM, and CMX. Similarly, the analysis of MT around implants also showed a significant increase for all the investigated treatment groups, compared with untreated sites. No other significant differences were noted among the other treatment groups of (VCMX, hADM, and pADM).

For the qualitative assessment of emerging technologies for bone regeneration, early investigational studies have been conducted evaluating the use of computer aided personalized scaffold design and manufacturing, stem cell transplantation, stem cell secretome delivery, and artificial intelligence. To date, there are limited controlled studies evaluating these approaches but the results of studies which have been conducted demonstrate safety, efficacy, and favorable patient‐reported outcomes. Clinical translation of these emerging, personalized technologies for hard tissue regeneration will largely depend upon feasibility and cost effectiveness.

**Conclusions:**

For the treatment of dento‐alveolar deficiencies within the hard or soft tissue structures around teeth and implants, we found that autogenous soft tissue grafts as well as soft tissue grafting substitutes are generally effective to yield a significant increase in the soft tissue thickness of the treated sites. In the regeneration of hard tissue, emerging, personalized strategies have demonstrated promising early outcomes, yet additional longitudinal, controlled studies are required before these modalities can be considered for widespread clinical adoption.

**Plain language summary:**

Dentists and researchers are looking to find new ways to help the gums and bone around teeth and dental implants heal and grow back. In this review, we looked at 78 studies that tested different methods for improving gum tissue thickness as well as rebuilding the bone that supports teeth and implants. We found that using a patient's own gum tissue is still the most effective way to thicken and strengthen the gums. However, newer materials made from collagen or donated tissue can work almost as well and are easier for patients because they do not require tissue to be taken from the mouth. Around dental implants, all of these options helped increase gum tissue thickness, which may lower the risk of future disease and improve the look and long‐term success of treatment. For rebuilding bone, early research on stem cells, 3D‐printed materials, and products that help the body to heal on its own looks very promising, but larger studies are needed to confirm the results. Overall, this research shows that regenerative dentistry is moving toward more comfortable, less invasive, and more personalized treatments that can improve healing, appearance, and long‐term stability.

## 1 INTRODUCTION

Regenerative therapies play a central role in modern periodontics and implant dentistry, aiming to restore lost structures and to reestablish both function and esthetics in cases of hard and soft tissue deficiencies. Although extensively explored, the long‐term outcomes of regenerative therapies remain variable and are influenced by a combination of patient‐specific factors and the complexity of the clinical presentation.^1^, ^2^ Also, soft and hard tissue deficiencies around teeth and dental implants differ not only in their morphology, but also their etiology, biological potential for healing, and esthetic challenges.^3^ As a result, standard treatment protocols often fall short when applied across a broad range of cases without accounting for individual variability. In addition, the field is experiencing a shift toward personalized regenerative strategies, supported by emerging technologies that allow clinicians to tailor interventions to each patient's specific anatomical and biological profile.^4^, ^5^, ^6^ Digital diagnostics,^7^ ultrasonographic tissue imaging,^8^, ^9^, ^10^, ^11^ AI‐assisted treatment planning,^12^, ^13^, ^14^, ^15^ and novel scaffold designs^16^, ^17^, ^18^ are reshaping the approach to regeneration—enabling more predictable, biologically sound, and less invasive procedures.

The steadily increasing use of soft tissue graft substitutes represents a major development in regenerative therapy. These biomaterials offer several advantages over autogenous grafts, including unlimited availability, the elimination of donor site morbidity, shorter surgical time, and often, improved patient acceptance.^19^, ^20^, ^21^ Based on their origin, soft tissue substitutes can be categorized as allogeneic, xenogeneic, or synthetic, and further divided by their composition and cellular activity into dermal matrices, collagen matrices, and cellular or acellular constructs.^22^, ^23^, ^24^ Among these, human acellular dermal matrices (hADM) have been used widely for decades in medicine and are now increasingly applied in dentistry.^25^, ^26^ These matrices act as biologically compatible scaffolds, supporting cellular migration and revascularization when used in bilaminar techniques for soft tissue augmentation or root coverage.^27^, ^28^

Similarly, xenogeneic alternatives, including porcine‐derived dermal matrices (pADM) and collagen‐based matrices, have gained traction due to their structural integrity and biological performance.^18^, ^28^, ^29^ The introduction of the second‐generation collagen matrices (CMX) has further expanded the options and approaches to regenerative therapies.^30^, ^31^ Characterized by a porous, cross‐linked structure, CMX support angiogenesis and mesenchymal cell infiltration, enabling scaffold‐guided tissue regeneration with significantly enhanced volume stability during early healing.^32^, ^33^, ^34^, ^35^, ^36^ Preclinical studies have demonstrated that the CMX not only integrate fully with host tissues but also promote favorable cellular responses, making it a viable option for both soft tissue augmentation and biologic delivery platforms, particularly when used with biologics and bioactive mediators.^32^, ^33^, ^37^, ^38^

Hard tissue regeneration around teeth and as part of implant therapy has also evolved significantly in recent years with the convergence of scaffold engineering, stem cell therapy, and personalized biologics. While the autogenous bone graft remains the gold standard due to its osteogenic potential, donor‐site morbidity, technique sensitivity, and unpredictable resorption have led to the emergence of more minimally invasive yet equally biologically responsive alternatives. These include acellular and cellularized scaffolds, bioactive carriers, and biologic modifiers, many of which are now under clinical investigation. While promising for improved and more predictable regenerative outcomes, these more personalized, emerging technologies for hard tissue regeneration have not been studied clinically as extensively as some of the aforementioned soft tissue regenerative modalities.

Taken together, these advancements and emerging technologies in soft and hard tissue regeneration point toward a more customized, biologically driven, and patient‐centered approach to regenerative dentistry. This article aims to explore the intersection of emerging technologies and personalized regenerative strategies, with a focus on their role in improving the clinical outcomes of soft and hard tissue therapies around natural teeth and dental implants. Specifically, through a synthesis of the current evidence, we aim to examine the application of such emerging technologies for the augmentation of soft and hard tissue defects around teeth and as part of implant therapy, evaluate their efficacy and effectiveness, and compare their clinical utility. By expanding on the unique contributions of each innovation, we seek to provide a practical framework for the integration of precision medicine and contemporary regenerative protocols into daily clinical practice.

## 2 MATERIALS AND METHODS

### 2.1 Protocol registration and reporting format

The protocol for this study was registered in the PROSPERO database^39^ (identification number CRD42025635583). The manuscript has also been prepared following the Cochrane Collaboration guidelines^40^ and is reported in accordance with the Preferred Reporting Items for Systematic reviews and Meta‐Analysis Extension (PRISMA) statement for Systematic Reviews incorporating network meta‐analyses for health care interventions.^41^, ^42^

### 2.2 Objectives

The goal of this systematic review was to address the previously stated focused questions regarding the availability and outcomes of emerging technologies and personalized strategies for improving hard and soft tissue defects around natural teeth and dental implants.

### 2.3 Population, intervention, comparison, outcome, study design (PICOS) question

As the focus of this review was on regenerative strategies for both soft and hard tissue augmentation, two separate frameworks were utilized to guide the literature assessment.

The following PICOS framework was used to guide the electronic and manual search of the literature towards assessing the focused question on soft tissue strategies:

- **Population (P)**: Patients (human adults) presenting with at least one natural tooth, or dental implant with a soft or hard tissue defect (at least one site in the oral cavity) that requires an augmentation procedure, with the objective of achieving regenerative outcomes.
- **Intervention (I)**: Employment of any professionally administered emerging technologies and/or personalized strategies which can lead to regeneration of periodontal or peri‐implant soft tissue thickness. The therapies could be conducted for enhancing soft and/or hard tissue thickness, volume or phenotype.
- **Comparison (C)**: All suitable comparisons among the included interventions and studies were explored.
- **Outcomes (O)**: Relative to the assessment of soft tissue regeneration around teeth, studies must have provided information on buccal soft tissue volume/thickness (either clinically or digitally) to be included, as this served as the primary outcome. Patient‐level outcomes (when available) such as intra‐operative discomfort/pain, post‐operative discomfort/pain, patient satisfaction, effect on quality of life, and treatment time were also explored.
- **Study design (S)**:
  - All prospectively conducted and controlled randomized interventional studies on human adults (whether split‐mouth or parallel‐design), with a minimum of 10 patients in the original trial, and at least a 12‐month post‐surgical follow‐up for teeth, and at least 6 months for dental implants were considered.
  - Studies had to have been published in a peer‐reviewed journal, and for a quantitative assessment of the therapeutic protocols and treatment effects only data from randomized controlled clinical trials (RCTs) with a defined protocol were utilized.

The following PICOS framework was used to guide the search of the literature towards assessing the focused question on hard tissue strategies:

- **Population (P)**: Patients (human adults) presenting with at least one natural tooth, dental implant, or oral and maxillofacial defect that requires a hard tissue augmentation procedure, with the objective of achieving regenerative outcomes.
- **Intervention (I)**: Employment of any professionally administered emerging technologies and/or personalized strategies which can lead to regeneration of periodontal or peri‐implant hard tissues, as well as for implant site development and/or congenital defects. The therapies could also be conducted for enhancing soft or hard tissue thickness, volume or phenotype.
- **Comparison (C)**: All suitable comparisons among the included interventions and studies were explored.
- **Outcomes (O)**: For assessment of the emerging technologies and personalized approaches for bone augmentation, any prospective research which focused on the above outcomes was considered.
- **Study design (S)**:
  - All prospectively conducted interventional studies on human adults, with an appropriate protocol were considered.
  - Studies had to be published in a peer‐reviewed journal.

### 2.4 Eligibility criteria, information sources, and search strategy

Prospectively conducted human clinical trials on the treatment of hard and soft tissue deformities/defects, as well as for implant site development and congenital defects without any restrictions on language, geographic location, or source were included, given they adhered to the above‐mentioned outcomes, interventions, and study design.

Studies that did provide a clear protocol for the performed augmentation procedure (i.e. those that had a mixture of bone and soft tissue augmentation for a defect without providing distinguishable details) were excluded. In addition, relative to soft tissue augmentation, RCTs with a follow‐up time of less than 12 months for teeth, and 6 months for implant were excluded, along with those studies treating only a specific population cohort (e.g., studies recruiting only certain individuals such as smokers or diabetics, etc.). Additional details on the eligibility criteria can be found in the Supplementary Appendix.

A detailed computerized and systematic literature search was conducted in the following electronic databases: The National Library of Medicine (MEDLINE via PubMed); EMBASE via OVID; the Cochrane Central Register of Controlled Trials; and Latin American & Caribbean Health Sciences Literature (LILACS), Web of Science, and Scopus. For examining unpublished trials, the grey literature, nonprofit reports, government research or other materials, were also electronically explored through searching in ClinicalTrial.gov and OpenGrey. Additional details are reported in the Supplementary Appendix.

### 2.5 Study selection and data retrieval

Two calibrated review authors (SB, HS) independently performed the selection process of the studies in duplicate. If needed, a third reviewer (L.T.) was consulted. Details are provided as a Supplementary Appendix.

### 2.6 Methodological quality and assessment of risk of bias

The assessment of methodological quality and “risk of bias” of the included studies was performed for RCTs on soft tissue regeneration, where data were to be utilized for quantitative assessment. This was done independently and in duplicate by two examiners (SB, HS), according to the recommended approach by the Cochrane collaboration group^43^ (Supplementary Appendix).

### 2.7 Synthesis of results and statistical methodology

For the quantitative analysis planned from RCT on soft tissue regeneration around teeth and implants, after transitivity assessment, a frequentist mixed‐modeling approach to network meta‐analysis^44^, ^45^, ^46^, ^47^ was utilized to model the aggregate data for the primary outcome of increased soft tissue thickness around teeth [gingival thickness (GT)], and mucosal thickness (MT) around implants. It was planned that the changes in the stated outcomes after surgical therapies, compared with a control group (flap therapy alone for both teeth and implants), and relative to other treatment modalities (any other surgical intervention).

Two sets of analyses (soft tissue thickness augmentation around teeth, and implants) were carried out. For all analyses and outcomes, all potentially relevant variables which may have had an influence on the outcomes were explored. In addition, the individual components of the rendered treatments (i.e. the specified regenerative therapies in each study arm) were modeled and analyzed. Similar to methodologies applied in previous work,^44^, ^47^, ^48^, ^49^ study arms were weighted by their effective treatment sample size (i.e. the number of treated defects per study arm) and clustered by publication cohort. For studies that utilized the same patient population (i.e. different follow‐up reports of the same original research), only one report with the most informative and complete data was utilized in the analyses.

Baseline demographics and clinical characteristics of the defects and patients were all accounted for in all models by inclusion of fixed covariates, and their influence on each outcome was assessed. Random effects were included to capture unique intercepts for study, study arms, as well as random slopes for study by time, and study arm by time.

The construction of the models was accomplished through testing a series of specifications of random and fixed effects via different model structures, utilizing mainly Akaike Information criterion (AIC) as evidence for the model that best fit the data.^50^ Confidence intervals (CIs) were produced, and a *p*‐value threshold of below 0.05 was set for statistical significance. The statistical analyses were performed by an author with experience in network meta‐analyses and linear mixed models (S.B.), using a specified software‡ and the statistical packages lme4,^51^ lmerTest,^52^ dplyr,^53^ and tidyr.^54^ The igraph^55^ and ggplot2^56^ packages were used for producing the geometry of the network plot to visualize the within study contrasts and the existing relationships among treatment arms.

### 2.8 Grading the certainty of evidence of the quantitative assessment of soft tissue regeneration

For the quantitative assessment of soft tissue regeneration around teeth and implants, we employed a modified approach to the Grading of Recommendations Assessment, Development, and Evaluation (GRADE) framework,^57^, ^58^ as described in a recent report to evaluate the certainty of evidence. The methods and criteria for this are detailed in the Supplementary Appendix.

### 2.9 Qualitative assessment of studies on personalized approaches and emerging technologies for bone tissue regeneration

For this section of the review a thorough assessment of the applicable literature was carried out to characterize studies based on the respective regenerative components and strategies of scaffold‐alone and acellular modalities, stem‐cell constructs and cellular enhancement approaches, and alternative cell sources. When available, data from prospective studies were grouped and described. Personalized constructs and digital strategies were also highlighted in narrative form. Due to the nature of the data in this realm and the high variability of the study designs and their respective outcome measures, a quantitative comparative assessment of these approaches was not feasible.

## 3 RESULTS

### 3.1 Scaffold‐based soft tissue regeneration

#### 3.1.1 Search results and study selection

The search strategy for studies on soft tissue regeneration yielded a total of 54 publications on teeth, and 24 for implants, which were included in the final analysis. This process is provided in depth in the text of the Supplementary Appendix, as well as Figure A1 of the Appendix which outlines this process.

**FIGURE 1:**
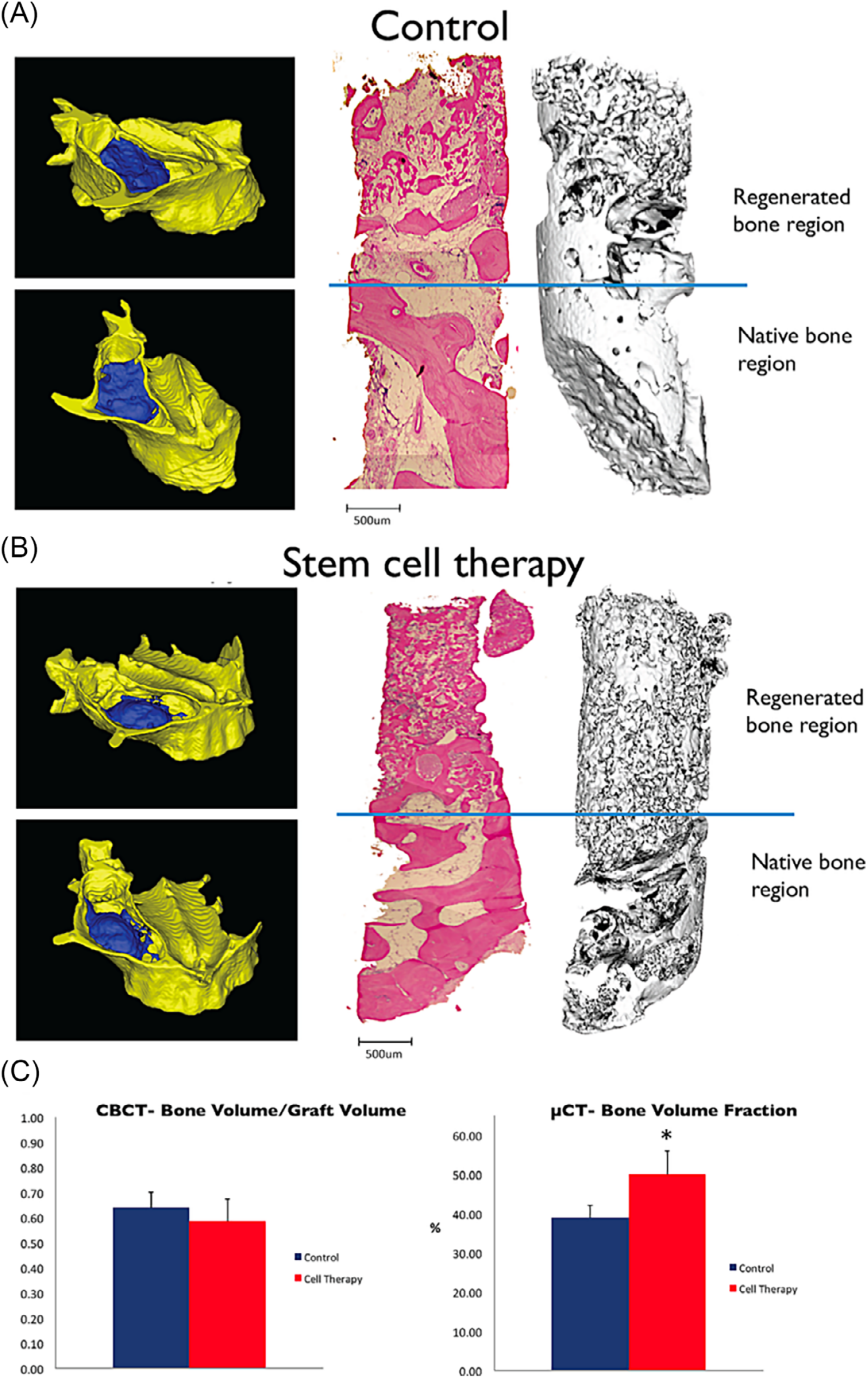
Representative images of 3D reconstructions of occlusal and lateral open views into the maxillary sinus cavity of the skull show the bone volume that was grafted (blue) in the control (A) and stem cell therapy (B) groups in severe bone defects. Histological and corresponding mCT images of bone biopsies harvested from the grafted regions of the two groups show a greater degree of mineralized bone tissue in the stem cell therapy group. (C) CBCT analysis of the bone volume/graft volume ratio (Bone Volume/Graft Volume) was no different between the control and stem cell therapy groups in treating severe defects; mCT analyses of the bone biopsies revealed that compared with the control, BVF was significantly higher in the stem cell therapy group in treating severe defects. (Reproduced with permission from Kaigler et al. 2015^59^).

#### 3.1.2 Characteristics of the included research, qualitative results, and risk of bias

Table 1 describes the characteristics of the articles included on natural dentition. As per the inclusion criteria, all references described an RCT, with the majority of them having been conducted at the University level, and most being parallel‐designed. All studies provided information on the primary outcome of soft tissue thickness augmentation at tooth sites [i.e. gingival thickness (GT)], with the included treatment arms of connective tissue graft (CTG), human acellular dermal matrix (hADM), porcine‐derived acellular dermal matrices (pADM), first generation collagen matrix (CMX), and “volume stable” collagen matrix (VCMX), as well as control sites of no soft tissue augmentation. The range of follow‐up for the included treatment arms was from 6 to 144 months.

**TABLE 1:** Study characteristics and interventions of the included randomized clinical trials on soft tissue augmentation around natural teeth.

Publication, reference	No. of centers, Setting, Original design, Funding	No. of arms included in NMA, No. of total study arms	Single/Multiple site treatment	Geographic location, Age (years), Inclusion of smokers	Treatment arm	Patients (*n*), Recessions (*n*)	Follow‐up timepoint (months)	Baseline GT (mm)	Follow‐up GT (mm)
Ahmedbeyli et al.^60^	1, University, Parallel, N	2, 2	Multiple	Asia, 29.2, none	FLAP	12, 24	12	0.71 ± 0.08	
					hADM	12, 24	12	0.75 ± 0.06	
Ahmedbeyli et al.^61^	1, University, Parallel, N	2, 2	Single	Asia, 29.04, none	FLAP	11, 11	12	0.72 ± 0.09	0.78
					hADM	11, 11	12	0.7 ± 0.11	1.39
Ahmedbeyli et al.^62^	1, University, parallel, N	2, 2	Multiple	Asia, NR, none	hADM	11, 28	12	0.69 ± 0.11	1.35
					hADM	11, 27	12	0.71 ± 0.09	1.35
Andrade et al.^63^	1, University, Split mouth, N	2, 2	Single	South America, NR, none	hADM	15, 15	12	0.48 ± 0.29	1.04 ± 0.44
					hADM	15, 15	12	0.55 ± 0.21	1.15 ± 0.45
Aroca et al.^64^	1, University, Split‐mouth, S	2, 2	Multiple	Europe, NR, none	CMX	22, 78	12	0.8 ± 0.2	1 ± 0.3
					CTG	22, 78	12	0.8 ± 0.3	1.3 ± 0.4
Ayub et al.^65^	1, University, Split‐mouth, S	2, 2	Single	South America, 45, none	hADM	15, 15	6	0.91 ± 0.3	1.36 ± 0.31
					hADM	15, 15	12	0.91 ± 0.3	1.45 ± 0.36
					hADM	15, 15	6	0.76 ± 0.28	1.28 ± 0.27
					hADM	15, 15	12	0.76 ± 0.28	1.34 ± 0.31
Barootchi et al.^1^ (9‐year follow‐up of Wang et al.^66^)*	1, University, Parallel, N	2, 2	Single	North America, 37.2, none	hADM	5, 5	3	1.2 ± 0.27	1.8 ± 0.44
					hADM	5, 5	6	1.2 ± 0.27	1.8 ± 0.27
					hADM	5, 5	12	1.2 ± 0.27	1.8 ± 0.27
					hADM	5, 5	108	1.2 ± 0.27	1.8 ± 0.27
					hADM	7, 7	3	1.42 ± 0.53	1.85 ± 0.62
					hADM	7, 7	6	1.42 ± 0.53	1.64 ± 0.62
					hADM	7, 7	12	1.42 ± 0.53	1.78 ± 0.48
					hADM	7, 7	108	1.42 ± 0.53	1.92 ± 0.53
Barootchi et al.^67^	1, University, Parallel, N	2, 2	Multiple	North America, 42.6, none	FLAP	17, 14	6	1.07 ± 0.37	1.25 ± 0.32
					FLAP	17, 14	144	1.07 ± 0.37	0.93 ± 0.26
					CTG	17, 13	6	1.05 ± 0.29	2.07 ± 0.61
					CTG	17, 13	144	1.05 ± 0.29	2.11 ± 0.61
					CTG	17, 16	6	0.9 ± 0.27	1.72 ± 0.29
					CTG	17, 16	144	0.9 ± 0.27	1.62 ± 0.67
Bittencourt et al.^68^	1, University, Split‐mouth, S	2, 2	Single	South America, 33.5, none	FLAP	17, 17	6	1.04 ± 0.26	1.07 ± 0.21
					FLAP	17, 17	30	1.04 ± 0.26	0.97 ± 0.2
					CTG	17, 17	6	1.01 ± 0.17	1.47 ± 0.43
					CTG	17, 17	30	1.01 ± 0.17	1.34 ± 0.34
Bittencourt et al.^69^	1, University, Split‐mouth, N	2, 2	Single	South America, 34, none	CTG	24, 24	12	0.89 ± 0.23	1.19 ± 0.31
					CTG	24, 24	12	0.97 ± 0.18	1.31 ± 0.41
Cairo et al.^70^	1, University, Parallel, N	2, 2	Multiple	Europe, 33.4, 2 patients (≤ 10 cig/day)	CTG	16, 36	6	0.73 ± 0.08	1.39 ± 0.18
					CTG	16, 36	12	0.73 ± 0.08	1.39 ± 0.18
				Europe, 35.1, 2 patients (≤ 10 cig/day)	FLAP	16, 38	6	0.76 ± 0.09	0.76 ± 0.1
					FLAP	16, 38	12	0.76 ± 0.09	0.76 ± 0.1
Cardaropoli et al.^71^	1, Private practice, Parallel, S	2, 2	Single	Europe, 35.63, none	CTG	18, 11	12	0.86 ± 0.39	2.09 ± 0.44
				Europe, 46, none	CMX	18, 11	12	0.82 ± 0.34	1.82 ± 0.51
Cardaropoli et al.^72^	1, Private practice, Parallel, N	2, 2	Multiple	Europe, 36.8, none	CMX	17, 58	12	0.89 ± 0.37	1.81 ± 0.48
				Europe, 39.9, none	FLAP	15, 54	12	0.81 ± 0.36	0.94 ± 0.36
Chen et al.^73^	1, University, Parallel, N	2, 2	Multiple	Asia, 35.33, none	CTG	12, 31	12	1.36 ± 0.45	1.7 ± 0.66
				Asia, 37.35, none	CTG	12, 28	12	1.19 ± 0.34	1.88 ± 0.5
Clementini et al.^74^	1, University, Parallel, N	2, 2	Single	Europe, 36.4, yes (<10 cig/day)	FLAP	20, 20	12	0.9 ± 0.1	
				Europe, 38.4, yes (<10 cig/day)	FLAP	20, 20	12	0.9 ± 0.2	
de Queiroz Cortes et al.^75^	1, University, Split‐mouth, N	2, 2	Single	South America, 32.8, none	FLAP	13, 13	6	1.05 ± 0.22	1.29 ± 0.2
					FLAP	13, 13	12	1.05 ± 0.22	1.23 ± 0.21
					hADM	13, 13	6	1.05 ± 0.27	1.75 ± 0.33
					hADM	13, 13	12	1.05 ± 0.27	1.65 ± 0.25
					FLAP	13, 13	24	1.05 ± 0.22	1.18 ± 0.21
					hADM	13, 13	24	1.05 ± 0.27	1.56 ± 0.27
de Melo Menezes et al.^76^	1, University, Split‐mouth, S	2, 2	Single	South America, 30.3, none	CMX	15, 30	18	0.83 ± 0.21	1.1 ± 0.4
				South America, 30.3, none	CTG	15, 30	18	0.89 ± 0.06	1.45 ± 0.41
Di Domenico et al.^77^	1, University, Parallel, S	2, 2	Multiple	Europe, 46.86, none	VXCM	22, 22	12	1.34 ± 0.48	1.57 ± 0.51
				Europe, 44.6, none	VXCM	20, 20	12	1.28 ± 0.46	1.64 ± 0.57
Elena et al.^78^	1, University, parallel, S	2,2	Multiple	Europe, 48.70, Yes (5%)	CMX	10, 58	12	1.09 ± 0.28	1.55 ± 0.61
				Europe, 48.39, Yes (5%)	CTG	10, 53	12	1.19 ± 0.29	1.97 ± 0.55
Franca‐Grohmann et al.^79^	1, University, Parallel, N	1, 2	Single	South America, 28.8, none	FLAP	15, 15	6	1.11 ± 0.16	1.44 ± 0.26
					FLAP	15, 15	12	1.11 ± 0.16	1.47 ± 0.29
Górski et al.^80^	1, University, Split‐mouth, N	2, 2	Multiple	Europe, 28.87, none	CTG	19, 67	12	1.25 ± 0.33	1.81 ± 0.92
				Europe, 28.87, none	CTG	19, 69	12	1.33 ± 0.47	1.93 ± 0.63
Jepsen et al.* ^81^	2, University, Split‐mouth, S	2, 2	Single	Europe, 44, yes (<10 cig/day)	FLAP	18, 18	12	0.96 ± 0.34	1.14 ± 0.38
					CMX	18, 18	12	0.93 ± 0.27	1.44 ± 0.4
					FLAP	18, 18	36	0.96 ± 0.34	1.11 ± 0.41
					CMX	18, 18	36	0.93 ± 0.27	1.52 ± 0.41
Koseoglu et al.^82^	1, University, Split‐mouth, S	1,2	Single	Asia, 45, none	CMX	11, 11	6	1 ± 0.31	1.26 ± 0.33
					CMX	11, 11	12	1 ± 0.31	1.09 ± 0.31
Kuka et al.^83^	1, University, Parallel, N	1,2	Multiple	Europe, 32.35, none	FLAP	12, 24	12	0.73 ± 0.07	0.8 ± 0.08
Leknes et al.^84^	1, University, Split‐mouth, S	1, 2	Single & Multiple	Europe, 38.4, yes (8 heavy smokers 20 cig/day)	FLAP	20, 20	6		
					FLAP	20, 20	12		
					FLAP	11, 11	72		0.8 ± 0.2
Macedo et al.^85^	1, University, Split‐Mouth, N	2, 2	Multiple	South America, 35, none	CTG	14, 36	16	1.44 ± 0.41	1.44 ± 0.5
Meza‐Mauricio et al.^86^	1, University, Parallel, N	2, 2	Multiple	South America, 38.1, none	CTG	20, 66	12	0.85 ± 0.25	1.53 ± 0.38
				South America, 36.3, none	pADM	21, 64	12	0.81 ± 0.23	1.26 ± 0.22
Molnár et al.^87^	1, University, Split‐mouth, S	2, 2	Multiple	Europe, NR, none	CTG	16, 78	108	0.86 ± 0.29	1.57 ± 0.35
				Europe, NR, none	CMX	16, 78	108	0.83 ± 0.26	1.49 ± 0.32
Ozenci et al. ^88^	1, University, Parallel, N	2,2	Multiple	Asia, 30.7, none	hADM	31, 10	12	0.82 ± 0.06	1.4 ± 0.07
				Asia, 30.7, none	hADM	27, 10	12	0.76 ± 0.06	1.38 ± 0.09
Paolantonio^89^	1, University, Parallel, N	1, 2	Single	Europe, 33.6, none	CTG	15, 15	12	0.8 ± 0.26	2.02 ± 0.62
Paolantonio et al.^90^	1, University, Parallel, N	1, 2	Single	Europe, 34.5, none	CTG	15, 15	12	0.81 ± 0.3	1.96 ± 0.42
				Europe, 34.5, none	hADM	15, 15	12	0.8 ± 0.36	1.8 ± 0.39
Pietruska et al.^91^	1, University, Split‐mouth, S	2, 2	Multiple	Europe, NR, none	CTG	15, 46	12	0.76 ± 0.31	1.86 ± 0.48
				Europe, NR, none	pADM	14, 45	12	0.82 ± 0.3	1.1 ± 0.37
Rakasevic et al.^92^	1, University, Split‐mouth, S	2, 2	Multiple	Europe, 30.5, 5	pADM	20, 62	12	0.61 ± 0.2	1.39 ± 0.44
				Europe, 30.5, 5	CTG	20, 52	12	0.69 ± 0.26	1.3 ± 0.38
Rebele et al.* ^93^	1, Private practice, Parallel and split‐mouth, N	2, 2	Single & Multiple	Europe, 37.9, none	CTG	14, 23	6		1.69 ± 0.4
					CTG	14, 23	12		1.63 ± 0.42
Rotundo et al.^94^	1, Private practice, Parallel, S	2, 2	Multiple	Europe, 31.4, none	CMX	12, 30	6	1.4 ± 0.7	1.6 ± 0.8
				Europe, 31.4, none	CMX	12, 30	12	1.4 ± 0.7	1.7 ± 0.7
				Europe, 38.1, none	FLAP	12, 31	6	1.5 ± 0.6	1.4 ± 0.6
				Europe, 38.1, none	FLAP	12, 31	12	1.5 ± 0.6	1.2 ± 0.5
Santamaria et al.^95^	1, University, Parallel, N	1,2	Single	South America, 40.5, no	CTG	20, 20	6	1.26 ± 0.3	2.09 ± 0.33
					CTG	20, 20	12	1.26 ± 0.3	2.04 ± 0.34
Neves et al.^96^ (follow‐up of Santamaria et al.)^95^	1, University, Parallel, N	2, 2	Single	South America, 38.2, none	CTG	20, 20	6	1 ± 0.4	1.7 ± 0.3
					CTG	20, 20	12	1 ± 0.4	1.6 ± 0.57
					CTG	20, 20	24	1 ± 0.4	1.7 ± 0.4
				South America, 38.9, none	CTG	19, 19	6	1 ± 0.3	2 ± 0.4
					CTG	19, 19	12	1 ± 0.3	1.9 ± 0.4
					CTG	19, 19	24	1 ± 0.3	2 ± 0.4
Skierska et al.^97^	1, University, Split‐mouth, N	2,2	Multiple	Europe, 32.54, none	CTG	24, 133	12	1.7 ± 0.75	2.54 ± 0.67
Stefanini et al.* ^98^	6, University, Split‐mouth, S	2, 2	Single	Europe, 39.5, yes (<10 cig/day)	FLAP	45, 45	6	0.89 ± 0.34	1.23 ± 0.46
					FLAP	45, 45	12	0.89 ± 0.34	1.16 ± 0.37
				Europe, 39.5, yes (<10 cig/day)	CMX	45, 45	6	0.89 ± 0.28	1.48 ± 0.46
					CMX	45, 45	12	0.89 ± 0.28	1.41 ± 0.46
Tavelli et al.^99^	1, University, Parallel, S		Multiple	North America, 52.1, none	hADM	9, 33	6	1.06 ± 0.45	1.46 ± 0.69
		2,2			hADM	10, 34	6	1.15 ± 0.34	1.51 ± 0.61
					hADM	9, 33	144	1.06 ± 0.45	1.28 ± 0.53
					hADM	10, 34	144	1.15 ± 0.34	1.34 ± 0.47
Tavelli et al.^38^	1, University, parallel, Y	2, 1	Multiple	North America, 40.9, 1 (≤10 cig/day)	VXCM	15, 44	6	0.84 ± 0.27	1.38 ± 0.33
Trivedi et al.^100^	1, University, Split‐mouth, N	2, 2	Multiple	Asia, 35.76, none	FLAP	25, 81	12	1.3 ± 0.11	1.59 ± 0.6
Uzun et al.^101^	1, University, Parallel, N	1, 2	Multiple	Asia, 40.3, none	CTG	18, 51	6	1.32 ± 0.29	1.97 ± 0.47
				Asia, 40.3, none	CTG	18, 51	12	1.32 ± 0.29	1.85 ± 0.5
Wang et al.^102^	4, University, Parallel, S	2, 2	Single	North America, 43, none	hADM	38, 38	12	1.12 ± 0.87	1.73 ± 0.59
				North America, 47.4, none	hADM	42, 42	12	0.74 ± 0.86	1.79 ± 0.62
Wang et al.* ^66^	1, University, Parallel, S	2, 2	Single	North America, 37.2, none	hADM	10, 10	12	0.9 ± 0.77	1.5 ± 0.33
				North America, 46.4, none	hADM	10, 10	12	0.95 ± 0.96	1.5 ± 0.58
Yavuz et al.^103^	1, University, Split‐mouth, Y	2, 2	Multiple	Asia, 37.3, none	CTG	12, 29	12	1.11 ± 0.41	1.5 ± 0.33
Yilmaz et al.^104^	1, University, parallel, N	2, 2	Multiple	Asia, 43.55, none	CTG	25, 87	12	0.71 ± 0.21	1.36 ± 0.45
Zucchelli et al.^20^	1, University, Parallel, N	2, 2	Single	Europe, 34.7, yes (<10 cig/day)	CTG	25, 25	12	0.71 ± 0.15	1.32 ± 0.22
					CTG	25, 25	12	0.75 ± 0.15	1.55 ± 0.21
Zucchelli et al.^105^	1, University, Parallel, N	2, 2	Single	Europe, 32.2, yes (<10 cig/day)	CTG	25, 25	12	0.84 ± 0.22	1.64 ± 0.26
				Europe, 34.2, yes (<10 cig/day)	FLAP	25, 25	12	0.77 ± 0.32	1.2 ± 0.35
Zucchelli et al.^106^	1, University, Parallel, N	2, 2	Single	Europe, NR, yes (<10 cig/day)	CTG	25, 25	12	0.43 ± 0.11	1.01 ± 0.28
					CTG	25,25	12	0.42 ± 0.11	1.2 ± 0.31
Zucchelli et al.^107^	1, University, Parallel, N	2, 2	Single	Europe, NR, yes (<10 cig/day)	CTG	30, 30	12	0.75 ± 0.15	1.47 ± 0.16
					CTG	30, 30	12	0.72 ± 0.13	2.11 ± 0.17
Zuhr et al.* ^108^	1, Private practice, Parallel and split‐mouth, N	2, 2	Single & Multiple	Europe, 37.9, none	CTG	14, 23	6		1.69 ± 0.4
					CTG	14, 23	12		1.63 ± 0.42
Zuhr et al.,^109^ Zuhr et al.^108^ and Rebele et al.^93^	1, Private practice, parallel, N	2, 2	Single & Multiple	Europe, 37.9, none	CTG	14, 23	12	1.32 ± 0.26	0.95 ± 0.41
				Europe, 37.9, none	CTG	15, 24	24	1.11 ± 0.26	0.95 ± 0.41
				Europe, 37.9, none	CTG	10, 22	60	0.95 ± 0.41	0.95 ± 0.41

The articles are in alphabetical order. NA, not available. NR, not reported. N/S in Funding describe Non‐funded, or a Supported clinical trial. mm, millimeter. CTG, connective tissue graft; CMX, first generation collagen matrix; Cig/day: cigarettes per day; FLAP, flap therapy (as control treatment); hADM, human acellular dermal matrix; pADM, porcine‐derived dermal matrix; RCT, randomized clinical trial; VCMX, “volume‐stable” collagen matrix. Final GT refers to the measurement at the last follow‐up recall of the corresponding study (in case of multiple timepoints).

^*^Articles pertaining to the same study patient population

The characteristics of the included studies on implants is presented in Table 2. Among the included RCTs, most were conducted in Europe, and similar to the studies on natural dentition, most were conducted at a university level, and the majority were university‐sponsored. The investigated treatment arms were CTG, hADM, pADM, CMX, VCMX, as well as no thickness augmentation (which served as the negative control). The range of follow‐up for the included treatment arms was from 6 to 48 months.

**TABLE 2:** Characteristics of the included treatment arm studies around implants.

Publication, reference	Study design	No. of centers, Geographic location, Setting, Funding	Treatment	Timing of intervention	Participant age (years), No. Male/Female, Inclusion of smokers	Final follow‐up time (months)	Patients (*n*), Implant sites (*n*)	Baseline MT	Final^*^ MT
Abdelwahab et al.^110^	RCT	1, Africa, University, N	CTG	At implant placement	42 (3/7), no	12	10, 10	1.9 ± 0.2	3.5 ± 0.5
Anderson et al.^111^	RCT	1, North America, University, sponsored	hADM	Delayed	49, NA, yes	3, 6	6, 6	2.25 ± NA	3.5 ± NA
			CTG	Delayed	49, NA, yes	3, 6	7, 7	2.14 ± NA	3.07 ± NA
Cairo et al.^112^	RCT	1, Europe, University, sponsored	CMX	At second stage	50.3, 10/20, yes	3, 6	30, 30	2.1 ± 0.6	3 ± 0.7
			CTG		48.3. 6/24, yes	3, 6	30, 30	2.1 ± 0.6	3.4 ± 0.6
Clem et al.^113^	RCT	5, North America, private practice, N	VXCM	Delayed	53.16, (18/13), no	12	31, 31	NR	0.93 ± 0.8
	RCT		CTG	Delayed	57.76, (20/13), no	12	33, 33	NR	1.1 ± 0.51
Cosyn et al.^114^	RCT	6, Europe, University, Sponsored	CTG	At implant placement	50.1 (15/15), no	12	29, 29	NR	26.35 mm3
			VXCM		48.2 (16/14), no	12	29, 29	NR	16.92 mm3
D'Elia et al.^115^	RCT	1, Europe, University, sponsored	CTG	At implant placement	50.7, 7/8, yes	6, 12	15, 15	3.1 ± 1.7	3.73 ± 1.13
De Bruyckere et al.^116^	RCT	1, Europe, University, sponsored	CTG	At implant placement	48, 12/9, no	12	21, 21	1.7 ± 0.76	2.68 ± 0.67
Hamdy et al.^117^	RCT	1, Africa, University, NR	VXCM	At implant placement	32 (8/2), Yes	9	10, 10	2.1 ± 0.32	2.94 ± 0.31
			CTG		35.5 (6/4), yes	9	10, 10	2.1 ± 0.32	3.87 ± 0.91
Happe et al.^118^	RCT	1, Europe, private practice, Industry sponsored	pADM	At immediate implant placement	48.9 years, NR, yes	12	10, 10		0.55 ± 0.33
			CTG		48.9 years, NR, yes	12	10, 10		0.6 ± 0.49
Huber et al.^119^	RCT	1, Europe, University, sponsored	CTG	Prior to abutment connection	43.4, 4/6, yes	6, 12	10, 10	2.7 ± 0.4	3.1 ± 1.3
			VXCM	Prior to abutment connection	44.1, 3/7, no	6, 12	10, 10	3.2 ± 0.8	2.8 ± 0.7
Kassis and Aboud ^120^	RCT	1, Asia, University, NR	CTG	At second stage	NR, 9/11, NR	12	20, 20	0.97 ± 0.22	1.62 ± 0.18
Lazzari et al.^121^	RCT	1, South America, University, NR	CTG	At implant placement	46.5, 9/11, NR	12	20, 20	2.36 ± 0.94	3.23 ± 0.77
			No soft tissue augmentation		47.6, 15/7, NR	12	22, 22	2.30 ± 0.98	2.30 ± 0.70
Lee et al.^122^	RCT	1, North America, University & private practice, sponsored	CTG	At immediate implant placement	61.53, 8/7, yes	6	15, 15	1.24 ± 0.25	2.10
					61.53, 8/7, yes	12	15, 15	1.24 ± 0.25	2.04
			hADM		63.53, 7/8, no	6	15, 15	1.34 ± 0.25	2.31
					63.53, 7/8, no	12	14, 14	1.34 ± 0.25	2.20
Migliorati et al. ^123^	RCT	1, Europe, University, NR	CTG	At immediate implant placement	47.5, 12/12, Yes	12	24, 24	1.1 ± 0.6	1.8 ± 0.8
			CTG		47.5, 12/12, Yes	24	24, 24	1.1 ± 0.6	1.5 ± 0.8
			No soft tissue augmentation		47.5, 13/10, Yes	12	23, 23	1.6 ± 0.6	1.1 ± 0.5
			No soft tissue augmentation		47.5, 13/10, Yes	24	23, 23	1.6 ± 0.6	1 ± 0.5
Oh et al.^124^	RCT	1, North America, Private practice, self‐supported	No soft tissue augmentation	NA	66, 3/4, no	48	7, 8		
			CTG	At second stage	48.87, 5/3, yes	6, 12	8, 8	1.22 ± 0.27	
			No soft tissue augmentation	NA		12	32, 32		
Panwar et al.^125^	RCT	1, Asia, University, Government sponsored	hADM	At immediate implant placement	32.5, NR, no	6	10, 10	0.5 ± 0.094	0.56 ± 0.107
			CTG		31.9, NR, no	6	10, 10	0.45 ± 0.108	0.57 ± 0.082
Puzio et al.^126^	RCT	1, Europe, University, sponsored	CMX	At second stage	42.1, 5/10, yes	3, 12	15, 15	1.21 ± 0.49	2.1 ± 0.5
			CTG	At second stage	41.1, 9/6, yes	3, 12	15, 15	1.15 ± 0.4	2.68 ± 0.96
			No soft tissue augmentation	NA	43.3, 6/9	3, 12	15, 15	1.39 ± 0.7	2.1 ± 0.7
Puzio et al.^127^	RCT	1, Europe, University, sponsored	CMX	At second stage	42.1, 5/10, yes	12	15, 15	1.21 ± 0.49	2.1 ± 0.5
			CTG	At second stage	41.1, 9/6, yes	12	15, 15	1.15 ± 0.4	2.68 ± 0.96
			No soft tissue augmentation	NA	43.3, 6/9, yes	12	15, 15	1.39 ± 0.7	2.1 ± 0.7
Surdiacourt et al.^128^, Cosyn et al.^114^	RCT	6, Europe, University, Sponsored	CTG	At implant placement	50.1, 15/15, no	36	29, 29	NR	0.83
			VXCM		48.2, 16/14, no	36	29, 29	NR	0.48
Tavelli et al. ^129^	RCT	1, North America, N	CTG	Delayed	46.9, 8/6, yes	6, 12	14, 14	1.18 ± 0.40	2.65 ± 0.50
			CTG	Delayed	47.1, 8/6, yes	6, 12	14, 14	1.42 ± 0.42	2.44 ± 0.34
Thoma et al.^130^	RCT	1, Europe, University, sponsored	CTG	NA	43.4, NA, yes	6, 12, 36	9, 9	2.7 ± 0.4	3.8 ± 1.5
			VXCM	NA	44.1, NA, no	6, 12, 36	8, 8	3.2 ± 0.8	3.6 ± 1.5
Wiesner et al.^131^	RCT	1, Europe, Private practice, NA	CTG	At implant placement	39, 3/7, no	12	10, 10	2 ± 0.47	3.2 ± 0.42
			No soft tissue augmentation	NA	39, 3/7, no	12	10, 10	2.05 ± 0.5	1.9 ± 0.32
Zafiropoulos & John ^132^	RCT	1, Europe, University, supported	pADM	At implant placement	47.2, 9/5, yes	6	14, 14	1.13 ± 0.4	2.19 ± 0.36
			No soft tissue augmentation	NA	45.1, 9/4, yes	6	13, 13		

Abbreviations: Cig/day: cigarettes per day; CMX, first generation collagen matrix; CTG, connective tissue graft; FLAP, flap therapy (as control treatment); hADM, human acellular dermal matrix; mm, millimeter; NA, not available; NR, not reported; N/S in Funding describes Non‐funded, or a Supported clinical trial; pADM, porcine‐derived dermal matrix; RCT, randomized clinical trial; VCMX, “volume‐stable” collagen matrix.

* Final MT refers to the measurement at the last follow‐up recall of the corresponding study (in case of multiple timepoints).

The quality assessment and the “Risk of bias” report of the included RCTs is presented in detail in the Supplementary Appendix. The results of the Risk of Bias assessment for the RCTs are included in the quantitative analysis.

#### 3.1.3 Quantitative results and mixed‐model network meta‐analysis for soft tissue regeneration around teeth via bilaminar approaches

The results from the analyses revealed that all the explored treatment modalities led to a significant increase in gingival thickness compared with no augmentation (flap treatments alone), with CTG showing the highest estimate in the model (0.62 mm (95% CI[0.47, 0.77]), *p* < 0.001), followed by VCMX (0.57 mm (95% CI[0.21, 0.93]), *p* < 0.01), hADM (0.48 mm (95% CI [0.28, 0.67]), *p* < 0.01), pADM (0.47 mm (95% CI[0.15, 0.81]), *p* < 0.01), and CMX (0.33 mm (95% CI[0.17, 0.49]), *p* < 0.01). However, when CTG was used as the reference for comparison, the only treatment arms that showed significantly less gingival thickness were flap treatment alone (−0.62 mm (95% CI[−0.77, −0.48]), *p* < 0.001), as well as CMX (−0.28 mm (95% CI [−0.43, −0.14]), *p* < 0.001). No differences could be observed among the other treatment arms of hADM (−0.14 mm (95% CI[−0.34, 0.05], *p* = 0.14)), VCMX (−0.05 (95% CI[−0.71, 0.61]), *p* = 0.86), and pADM (−0.14 (95% CI[−0.43, 0.14]), *p* = 0.32).

Baseline GT was found to positively predict the final GT gain (0.49 mm (95% CI [0.22, 0.76], *p* < 0.001)), however individually, this variable did not show a significant interaction with the other treatment arms (i.e. baseline GT affects all treatments positively, and in the same manner). However, baseline values of KTW (0.019 (95% CI[−0.08, 0.12]), *p* = 0.7), and gingival recession (0.05 (95% CI[−0.05, 0.16]), *p* = 0.29), when present did not influence the outcomes. Time did not influence the model (−0.0009 (95% CI[−0.001, 0.00009]), *p* = 0.08).

Furthermore, the increase in GT was significantly associated with reduced gingival recession over time (−0.16 (95% CI [−0.31, −0.01]), *p* = 0.02), similarly without treatment group interaction. Lastly, an increase in GT was associated with a reduction in plaque index (PI) over time (−0.39 (95% CI [−0.64, −0.13], *p* < 0.01).

#### 3.1.4 Quantitative results and mixed‐model network meta‐analysis for soft tissue regeneration around implants via bilaminar approaches

Among the generated comparisons, compared with untreated sites as the control group, all the investigated treatment arms of CMX (0.68 mm (95% CI [0.42, 0.95]), *p* < 0.01), pADM (0.71 mm (95% CI [0.12, 1.29]), *p* = 0.02), VCMX (0.88 mm (95% CI [0.21, 1.54]), *p* = 0.01), hADM (0.98 mm (95% CI [0.62, 1.33]), *p* < 0.01), and CTG (1.12 mm (95% CI [0.91, 1.33], *p* < 0.001) were shown to significantly increase MT (in an increasing order of magnitude).

With CTG as the reference group, the only treatment arms that showed significant differences were untreated control sites (−1.12 mm (95% CI [−1.33, −0.91]), *p* < 0.001), and CMX (−0.44 (95% CI [−0.62, −0.26]), *p* < 0.01). The difference between the other intervention groups of ADM (−0.14 (95% [−0.17, 0.46]), *p* = 0.53), and XCM (−0.24 (95% CI [−0.56, 0.08]), *p* = 0.19) did not reach statistical significance.

The baseline MT also revealed a significant effect in the model (1.04 mm (95% CI [0.75, 1.33]), *p* < 0.001), which showed significant interactions with the treatment arms of CMX, and hADM). However, the baseline KT (−0.12 mm (95% CI [−0.54, 0.28]), *p* = 0.54) did not reveal a significant effect on MT outcomes.

The effect of time also fell below significance in the model (−0.01 (95% CI [−0.02, 0.0006], *p* = 0.07).

Regarding clinical parameters associated with peri‐implant health, it was found that MT augmentation can lead to a decrease in PD (−0.81 (95% CI [−1.25, −0.35], *p* = 0.02), however no other statistically significant effects could be observed with regard to changes in BOP (−0.52 (95% CI [−3.26, 2.21]), *p* = 0.72), or PI (−1.4 (95% CI [−7.87, 5.07], *p* = 0.7)).

#### 3.1.5 Certainty of evidence

Both outcomes demonstrated a high level of certainty. Gingival thickness gain with flap‐based techniques exhibited high certainty (*n* = 2972, low to moderate risk of bias), while mucosal thickness gain also showed high certainty (*n* = 851, low to moderate risk of bias). The certainty ratings were determined based on an evaluation of risk of bias, inconsistency, indirectness, and imprecision across the included studies. Notably, both outcomes showed no serious concerns in any domain (Table 3).

**TABLE 3:** Grading the certainty of evidence on the primary outcomes of gingival and mucosal thickness gain in soft tissue augmentation around teeth and implants. A modification to the GRADE approach was implemented.

	Certainty assessment									
Outcome variable	Estimate (95% CIs), *p* value	Study designs	Population (*N*) [Teeth/implants]	Minimum follow‐up (months)	ROB	Inconsistency	Indirectness^**^	Imprecision	Other considerations	Certainty
Gingival thickness (Ref: flap)	0.62 mm (95% CI [0.47, 0.77]), *p* < 0.001	RCTs	2972	12	Low to Moderate	Not Serious^*^	Not Serious	Not serious	None	High
Mucosal thickness (Ref: no treatment)	1.12 mm (95% CI [0.91, 1.33], *p* < 0.001	RCTs	851	6	Low to Moderate	Not Serious^*^	Not Serious	Not serious	None	High

Abbreviations: CTG, connective tissue graft; N, number; CI, confidence interval; ROB, risk of bias; Ref: reference.

* All possible inconsistency controlled for using mixed‐models regression analysis.

** All included studies directly addressed the focused question.

### 3.2 Evolution of surgical techniques for root coverage

The clinical performance of soft tissue grafting techniques has evolved in parallel with refinements in flap design and surgical access. Early CAF protocols demonstrated mean root coverage values in the range of 60%–75%,^133^, ^134^ while subsequent refinements—such as tension‐free flap advancement, split–full‐split elevation, and coronal positioning beyond the CEJ—consistently improved complete coverage rates to over 90% in favorable phenotypes.^135^, ^136^, ^137^

The development of tunneling approaches marked another significant step forward. The modified coronally advanced tunnel (MCAT) and microsurgical tunneling techniques demonstrated superior esthetic and patient‐reported outcomes compared with trapezoidal CAFs, while maintaining high levels of root coverage predictability.^138^, ^139^, ^140^ Clinical trials have confirmed that these minimally invasive approaches reduce morbidity while achieving outcomes comparable, or in some cases superior, to conventional CAF.^31^, ^141^

More recently, the tunneled coronally advanced flap (TCAF) has been described as an adaptable hybrid approach and a treatment philosophy which selectively combines CAF‐like access with tunneled flap preservation depending on site‐specific anatomy and papillary integrity.^4^ Prospective clinical studies of TCAF, including those integrating volumetric outcomes, have reported mean root coverage values approaching 95%–100%, with significant improvements in esthetic indices and volumetric tissue gain at mid‐term follow‐up.^17^ These findings underscore how progressive refinements in flap design and surgical access have been as critical to the advancement of regenerative therapy as the adjunctive use of grafting materials.

### 3.3 Scaffold‐based bone regeneration: a review on emerging, personalized, and minimally invasive technologies

Regenerating alveolar bone for periodontal and implant therapy has evolved significantly in recent years with the convergence of scaffold engineering, stem cell therapy, and personalized biologics. While autogenous bone remains the gold standard due to its osteogenic potential, donor‐site morbidity, and unpredictable resorption have catalyzed the development of novel alternatives. These include acellular and cellular scaffolds, bioactive carriers, and biologic modifiers, many of which have now entered the clinical arena. In what follows, we highlight key studies demonstrating the efficacy and translational promise of these strategies, with a focus on personalization and minimal invasiveness.

#### 3.3.1 Scaffold‐only and acellular strategies

The foundation of guided bone regeneration (GBR) relies on space maintenance, cell exclusion, and preferential cellular ingrowth, which is where scaffolds such as anorganic bovine bone mineral (ABBM), β‐tricalcium phosphate (β‐TCP), hydroxyapatite (HA), and alloplastic composites play an essential role. Shabaan et al. conducted a prospective trial on alveolar cleft bone reconstruction comparing autologous bone versus 3D‐printed calcium phosphate scaffolds, demonstrating comparable volumetric outcomes at 6 months, supporting scaffolds as valid substitutes in select indications.^142^

Similarly, Lavareda Corrêa et al. explored appositional reconstructions using allografts, with or without bone marrow aspirate concentrate (BMAC), revealing no statistically significant difference in bone fill, although BMAC groups trended toward yielding improved histomorphometric outcomes.^143^ Whitt et al. compared stem cell‐based allografts with standard allografts in sinus augmentation, reporting superior trabecular connectivity and bone density at 6 months in the stem cell group without differences being noted in tissue volume.^144^

Another acellular strategy is the use of the secretome and/or exosomes derived from stem cells for regeneration of hard tissue. This approach takes advantage of the paracrine properties of stem cells without the inclusion of the cellular component which could elicit an adverse immune response. The secretome of oral‐derived stem cells contains a number of cytokines which can act directly on other cells to promote their migration, proliferation, and differentiation. These paracrine effects can result directly in tissue regeneration via angiogenic, osteogenic, and neutrophic factors or can regulate the regenerative microenvironment through immunomodulation, anti‐inflammatory signaling, and anti‐microbial activities.^145^, ^146^, ^147^ The potent properties of the secretome and exosomes derived from oral‐derived stem cells has led to bioengineering of exosomes specifically designed for regenerative applications.^145^, ^146^, ^148^ These technologies utilize genetic engineering tools to generate small molecules, proteins, and nucleotides specific to various functions which can then be packaged into various biomaterials that can be used for their delivery.^147^, ^148^, ^149^, ^150^ Despite the promise of these cell‐free approaches, they are still in preclinical stages of development and there are a number of manufacturing and regulatory hurdles that need to be overcome for these emerging technologies to reach clinical application and adoption. These preclinical observations are consistent with a recent scoping review by Shanbhag et al., that emphasized the regenerative and immunomodulatory properties of bone‐derived MSC secretomes, highlights secretome‐based therapies as promising cell‐free alternatives that may overcome regulatory hurdles associated with direct stem cell transplantation, while retaining pro‐angiogenic and osteoinductive potential.^151^

#### 3.3.2 Stem cell–enhanced constructs

One of the most promising trends in bone regeneration involves mesenchymal stem cell (MSC)‐based therapies. In a seminal series of the first randomized, controlled clinical trials in the United States evaluating stem cell therapies for craniofacial bone regeneration, Kaigler et al.^59^, ^152^, ^153^ performed phase I/II studies using autologous, bone marrow‐derived stem cells (tissue repair cells, TRCs) for treating alveolar defects of various size and etiology. Treated defects were resultant of tooth extraction, maxillary sinus pneumatization, trauma, and congenital alveolar cleft and different cell carriers were used including gelatin sponges and β‐TCP. In these RCTs, taken together, regardless of the carrier, statistically significant improvements in bone volume, density, and implant integration over controls were noted with no safety related adverse events.^59^ Additionally, through histomorphometric analyses of bone core biopsies collected from the regenerated sites in these studies, enhanced vascularization, biomaterial degradation and bone quality were observed (Figure 1).

In addition to U.S.‐based phase I/II trials, parallel efforts in Europe have also demonstrated translational potential. Gjerde et al. reported a prospective clinical trial in which autologous bone marrow mesenchymal stromal cells combined with β‐TCP were used for severe mandibular ridge resorption. At 12 months, patients achieved stable bone formation with high implant survival, corroborating the safety and efficacy of MSC‐based constructs in craniofacial bone regeneration.^154^

In a long‐term retrospective follow‐up study, Asahina et al. confirmed the 8‐year stability of BMSC‐PRP–β‐TCP constructs in severely atrophic mandibles, validating the longevity and safety of these approaches.^155^ Gupta et al. compared MSCs with blood coagulum in sinus lift procedures in a split‐mouth RCT. Both groups achieved implant stability, however, MSCs showed slightly faster maturation and improved bone density at 6 months, though not statistically significant.^156^ Similarly, Payer et al. used autotransplanted tibial bone marrow aspirate in sinus lifts and observed greater histologic bone maturation compared with particulate bone alone.^157^

Intraoral MSC sources have also been explored. Khojasteh et al. combined buccal fat pad‐derived MSCs with ABBM for mandibular defects and demonstrated improved bone formation over ABBM alone.^158^

#### 3.3.3 Alternative stem cell sources

Expanding stem cell sources beyond bone marrow and fat, Feng et al. investigated small blood stem cells (SBSCs) embedded in gelatin microspheres for maxillary sinus augmentation. This injectable, minimally manipulated therapy produced significantly higher bone volume and density at 4 and 6 months compared with conventional xenografts.^159^

Chen et al. delivered periodontal ligament stem cells (PDLSCs) on collagen scaffolds to regenerate intrabony defects in periodontitis patients.^160^ Treated groups exhibited superior probing depth reduction and radiographic bone gain compared with controls. Sanchez et al. similarly applied PDLSCs with deproteinized bovine bone and showed increased cementum‐like tissue and mineralization histologically, supporting their osteogenic potential.^161^ Another sourcing of MSCs to treat periodontal intrabony defects was evaluated by Ferrarotti et al. in their use of autologous dental pulp‐derived stem cells (DPSCs) to treat these defects in patients with chronic periodontitis.^162^ In this case series, collagen sponges were used to deliver the DPSCs to defects and yielded significant clinical and radiographic improvements, including attachment gain and bone regeneration 1 year post‐operatively.

#### 3.3.4 Clinical outcomes and controlled trials

Baba et al.^163^ conducted a phase I/II clinical trial using autologous cultured MSCs with a biodegradable 3D woven‐fabric composite scaffold and PRP for patients with periodontitis, who required a surgical procedure for intrabony defects. MSCs were implanted in each periodontal intrabony defect, and patients were monitored for 36 months. The study found significant improvements in all clinical parameters (CAL, PD, linear bone growth), with no clinical safety concerns.

A randomized controlled study by Fatale et al. compared periosteal MSCs with β‐TCP scaffolds in lateral versus crestal sinus lifts and found improved histomorphometric parameters in the lateral approach group at 4 months.^164^

Ferrarotti et al. evaluated the efficacy of using micrografts containing autologous dental pulp tissue in combination with a collagen scaffold for the regeneration of human intrabony periodontal defects. In this RCT, the test group received dental pulp micrografts plus scaffold, while the control group received scaffold alone. After 12 months, the test group showed significantly greater CAL gain and PD reduction compared with controls, suggesting that autologous dental pulp micrografts may enhance periodontal regeneration, potentially due to the presence of progenitor cells and bioactive factors within the pulp tissue.^165^

As part of minimally invasive innovations, the study by Gupta et al. found that both MSC and blood‐coagulum sinus lifts led to primary stability of all implants, reinforcing the viability of biologically active but less invasive augmentation.^156^

More recently, Sanz et al. conducted a multicenter randomized clinical trial employing bone‐derived mesenchymal cells with a synthetic scaffold for alveolar ridge augmentation. The study demonstrated significant volumetric bone gain and favorable histomorphometric outcomes compared with scaffold alone, further supporting the adjunctive role of MSCs in enhancing scaffold‐mediated regeneration.^166^

#### 3.3.5 Personalized constructs and digital strategies

In the RCT conducted by Bajestan et al.^153^ evaluating stem cell therapy to treat highly morphologically variable cleft bone defects, despite safety being demonstrated, the clinical efficacy was highly variable.

**FIGURE 2:**
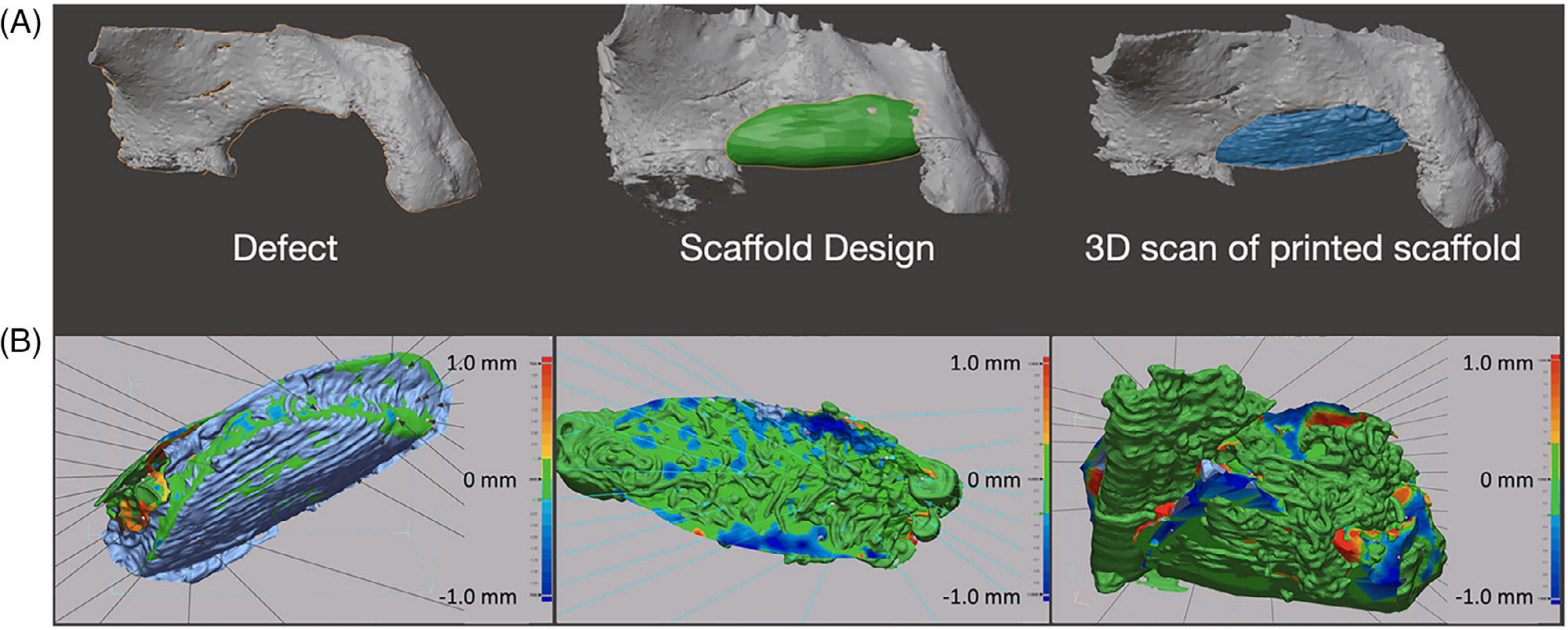
Fit of printed scaffolds to variably sized clinical defects. (A) Defect and digital design of defect‐specific scaffolds from patient 1. (B) The dimensions of the printed Osteoink scaffolds were compared with the 3D design using superimposed color mapping to determine accuracy of the precision‐fit of the scaffold with the alveolar defect. Models were superimposed by the best matching algorithm and the deviation of fit between the two model surfaces averaged among 30 different points. (Reproduced with permission from Anderson et al. 2022^167^).

**FIGURE 3:**
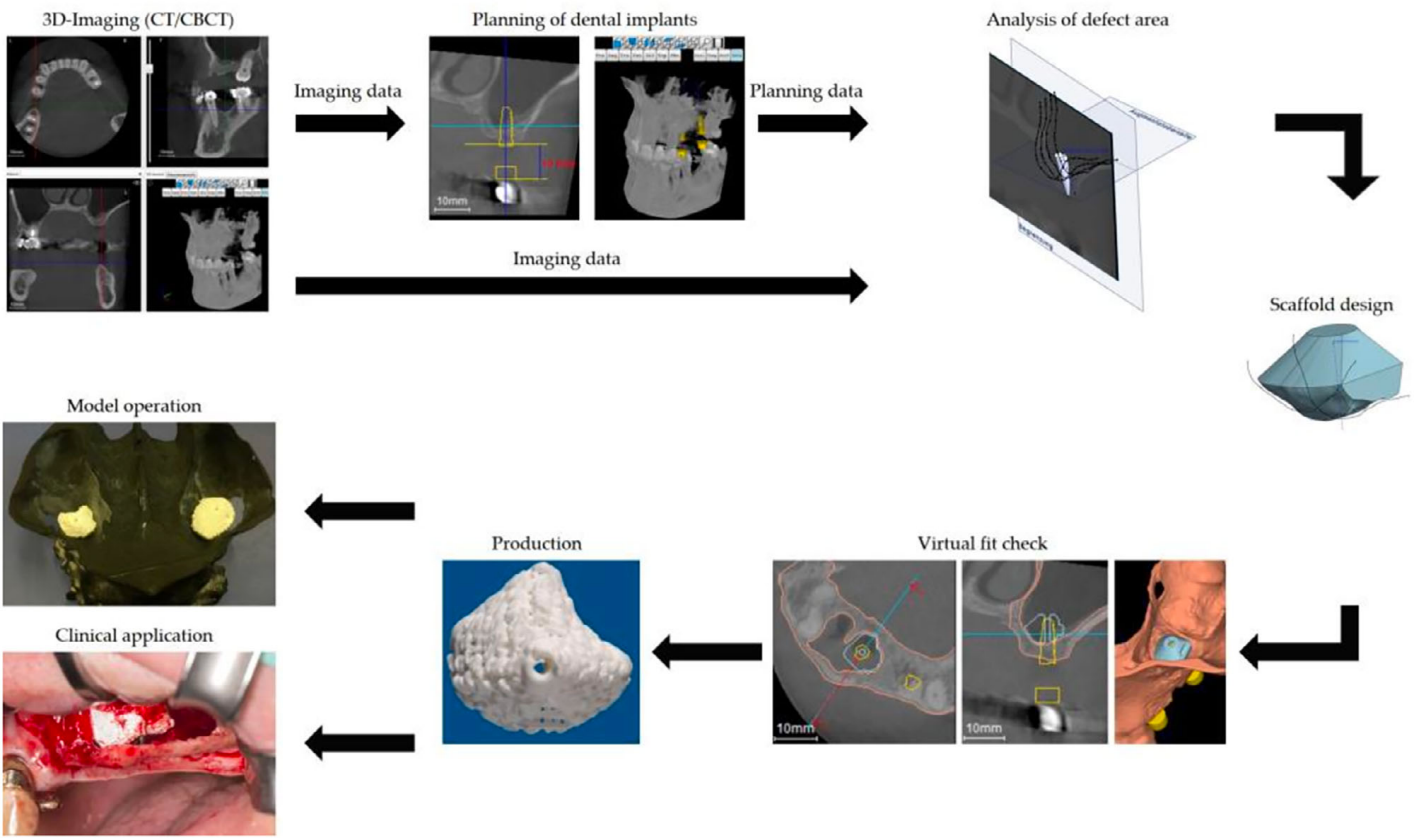
Schematic overview of the workflow in clockwise direction starting top left. Acquisition of the three‐dimensional radiographic data (upper left). Planning of the position of the dental implants with yellow contour indicating the planned implant and the corresponding sleeve (upper center). Analysis of the defect size and topography (upper right). Virtual design of the scaffold (center right). Virtual check of the designed scaffold with yellow contour indicating the planned implant and the corresponding sleeve, light blue contour indicating the planned scaffold and brown contour indicating the pristine bone in the transversal plane; coronary plane is indicated by the red arrows (lower right). Manufacturing of the scaffolds (lower center). Check of the designed scaffold in a three‐dimensional model of the defect situation (lower left, top). Clinical application of the scaffold in the patient (lower left, bottom). (Reproduced with permission from Schulz et al. 2023).^168^

One of the key contributing factors to the unpredictable clinical efficacy was the “one size fits all” approach to treating the highly individualized and variable nature of the bone defects between patients. Incorporating personalization into reconstructive bone regenerative therapy is an emerging technology that leverages upon the use of CAD‐CAM technology, enhanced biomaterials, and 3D bioprinting to yield personalized, precision‐fit scaffolds for stem cell delivery^153^, ^167^ (Figure 2). Schulz et al.^168^ applied defect‐specific three‐dimensionally plotted calcium‐phosphate scaffolds for personalized alveolar reconstruction prior to implant placement, demonstrating favorable bone regeneration and reduced postoperative morbidity. In this study, higher bone volumes were observed in the experimental groups that employed the CAD‐CAM technology and it is also important to note that in addition, patient discomfort post‐operatively was noted to be less in this group. Similarly, in a case report, Schultz et al. used a calcium phosphate paste to generate 3D printed scaffolds specific to defects of future implant sites in a patient^168^ (Figure 3). These proof‐of‐concept studies demonstrate efficacy, decreased surgical time, and increased patient comfort associated with the use of these technologies and represent a shift toward individualized scaffolding, which not only enhances fit and integration but may reduce complications.

Advancements in scaffold structure have also introduced smart biomaterials, as seen in newer polymeric composites explored by Asahina et al.,^155^ Feng et al.,^159^ and Baba et al.^163^ These materials have demonstrated early efficacy to improve implant osteointegration and they can also serve to deliver biologics, such as platelet concentrates, and can be tailored to enable defined, temporal release profiles of these biologics.

## 4 DISCUSSION

The present review offers a comprehensive synthesis of human trials evaluating different strategies for soft and hard tissue augmentation around teeth and dental implants.

### 4.1 Soft tissue augmentation around teeth

By conducting a mixed‐model based network meta‐analysis, our findings confirmed that all investigated interventions led to a significant increase in gingival thickness (GT) at natural teeth. Relative to soft tissue regeneration, the evaluated materials, autogenous connective tissue grafts (CTG) yielded the highest estimates, followed by VCMX. However, no statistically significant differences were observed between CTG and other soft tissue graft substitutes—including hADM, pADM, CMX, and VCMX—supporting the broader applicability of these materials in clinical practice, especially when minimally invasive or patient‐centered considerations take precedence. It should be noted that the analysis on soft tissue regeneration was intentionally limited to human randomized clinical investigations. While this criterion enhanced the clinical translatability of the findings, it also restricted the inclusion of several innovative tissue engineering strategies that are currently in exploratory or preclinical stages.

As regenerative dentistry moves toward more personalized and patient‐centered strategies, other dimensions—such as patient experience, post‐operative pain, and overall satisfaction—must be incorporated into the evaluation of treatment efficacy.^36^, ^169^, ^170^, ^171^, ^172^, ^173^ In this regard, CTG‐based procedures pose limitations.^19^, ^20^, ^21^ Long‐term follow‐up studies suggest that patient perception of pain during CTG harvesting can negatively influence their willingness to undergo similar procedures, even a decade after.^174^ These considerations have led to the development and growing adoption of soft tissue graft substitutes, which are generally better accepted by patients.^22^, ^26^, ^29^, ^175^, ^176^, ^177^ The present review demonstrates that significant GT gain can be achieved with a bilaminar approach using substitutes such as VCMX, CMX, hADM, and pADM. As such, graft selection should be guided by patient‐specific anatomical factors, such as the amount of keratinized tissue and defect complexity, as well as esthetic and comfort‐related expectations, rather than the assumption that autogenous grafts are universally superior.

### 4.2 Scaffolds and biomaterials in soft tissue regeneration

Tissue engineering in oral regeneration is predicated on the synergistic use of three foundational components: cells, signaling molecules, and scaffolds, supported by an adequate vascular supply.^178^, ^179^ These elements collectively aim to replicate or enhance the natural wound healing cascade. Scaffolds, in particular, serve as structural frameworks that guide tissue integration, facilitate cell migration, and may deliver biologically active cues.^6^, ^180^ In the realm of soft tissue regeneration, scaffolds can be derived from natural matrices (e.g., acellular dermal matrices, porcine‐derived collagen) or synthetic polymers.^22^, ^179^ Natural scaffolds such as acellular dermal matrices retain native extracellular matrix components that support fibroblast colonization and angiogenesis. Xenogeneic collagen matrices, often cross‐linked for volume stability, have shown favorable clinical and patient‐reported outcomes, albeit with some variability based on processing and origin.^22^, ^179^

**FIGURE 5:**
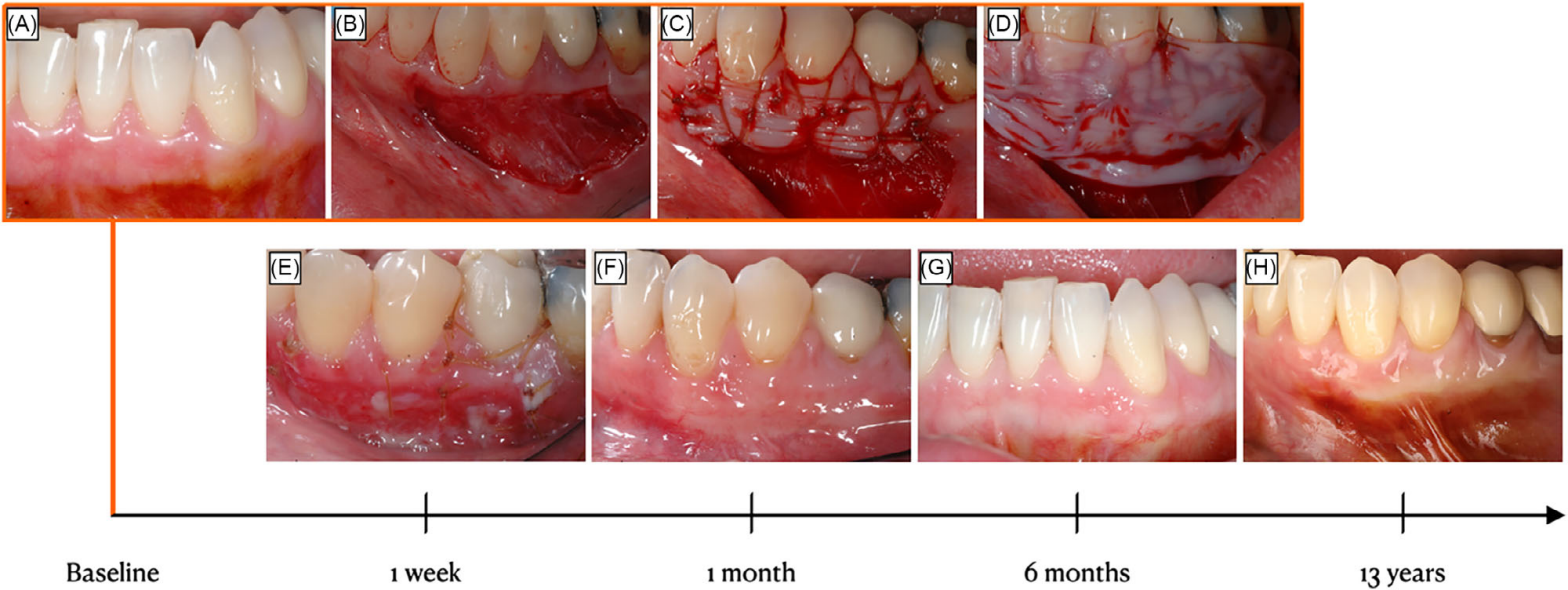
Non‐root coverage keratinized gingiva augmentation using a cell‐based tissue engineering strategy. A living cellular construct, characterized by allogeneic keratinocytes and fibroblasts from newborn foreskin seeded into a collagen membrane (Apligraft, Organogenesis, USA), was used in this case, that was part of a previously published clinical trial^184^. (A) Baseline. (B) Flap elevation. (C) Living cellular construct stabilized to apically to the canine and premolars. (D) An additional layer of the living cellular construct was applied over the graft. (E) 1‐week post‐op. (F) 1‐month post‐op. (G) 6‐month follow‐up. (H) Outcomes after 13 years. Note that Lugol's solution was used to discriminate the alveolar mucosa from the keratinized gingiva. (Reproduced with permission from Tavelli et al.^179^).

On the synthetic and bioengineering front, technologies such as 3D‐printed fiber‐guiding scaffolds, stem cell‐based constructs, and scaffold‐bioreactor systems are under active development. The use of additive manufacturing (AM) to generate personalized scaffolds has been described, with tailored mechanical and structural properties.^181^ Rasperini et al. demonstrated the first clinical application of a bioresorbable 3D‐printed scaffold in periodontal regeneration, opening the door to personalized regenerative solutions^182^ (Figure 4a and b). McGuire et al. successfully utilized a cell‐based tissue engineering strategy for non‐root coverage keratinized tissue augmentation as well (Figure 5).^183^ However, such innovations remain largely confined to case reports and early‐phase trials, with limited validation in large‐scale human studies.^181^

FIGURE 4(a) The first clinical application of a 3D‐printed bioresorbable scaffold for periodontal regeneration in 2015.^182^ (A) Baseline peri‐apical x‐ray. (B–C) clinical view of the tooth with periodontal infrabony defect. (D) Flap elevation. (E) Application of 24% EDTA for 2 min. (F) Polycaprolactone scaffold. (G) Scaffold soaked with recombinant human platelet‐derived growth factor‐BB (GEM21S, Lynch Biologics, USA). (H) Scaffold fixation to the alveolar bone. (I) Flap closure. (J) Healing after 2 weeks. (K) 1‐year post‐op. (Reproduced with permission from Rasperini et al.^182^ (b) The microcomputed tomography scan of the utilized customized multi‐compartments scaffold. (Reproduced with permission from Rasperini et al. 2015^182^).

**Figure:**
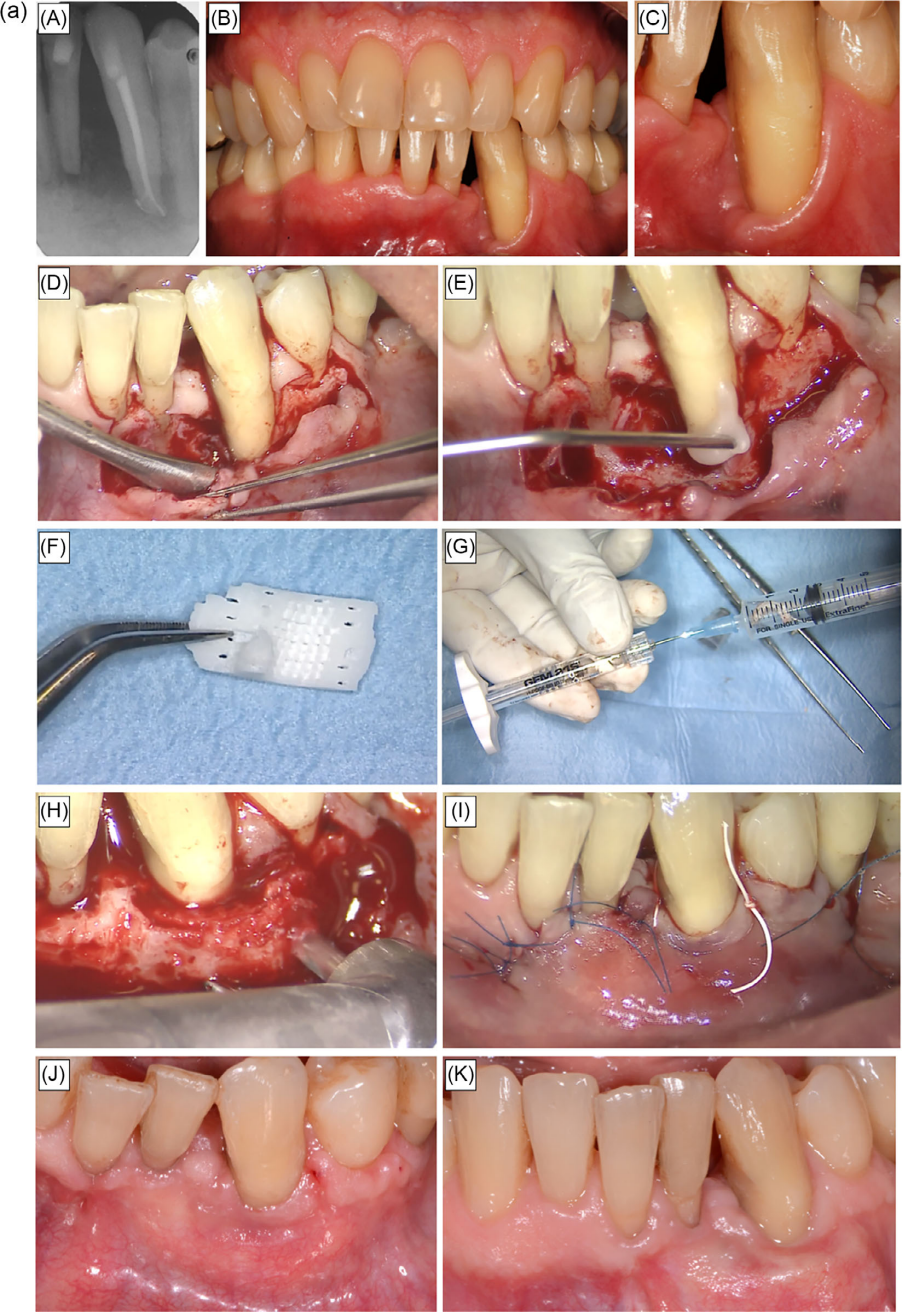

**Figure:**
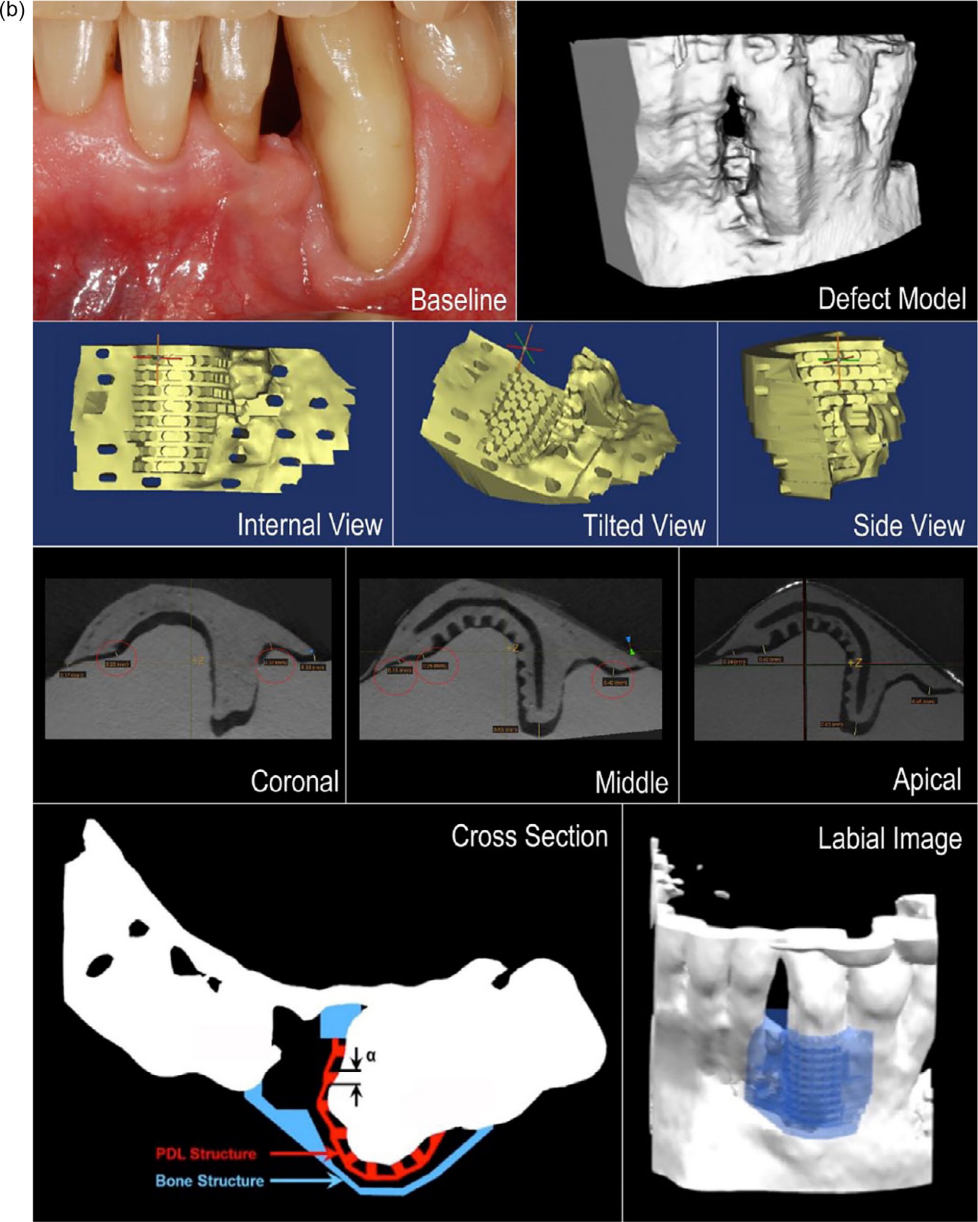

While these technologies are promising, most advanced scaffold systems incorporating gene delivery vectors, stem cells, or spatially patterned bioactive layers remain in the preclinical phase.^178^, ^185^ Regulatory, biological, and cost‐related hurdles must be overcome before widespread clinical adoption is feasible. Nonetheless, their trajectory indicates a strong potential to reshape the regenerative landscape in dentistry in the coming years. Importantly, a central theme emerging from both clinical trials and translational research is the emphasis on minimally invasive treatment strategies. Tissue engineering strategies that eliminate the need for secondary harvest sites and promote faster healing are therefore particularly relevant in the modern practice paradigm.^179^

**FIGURE 6:**
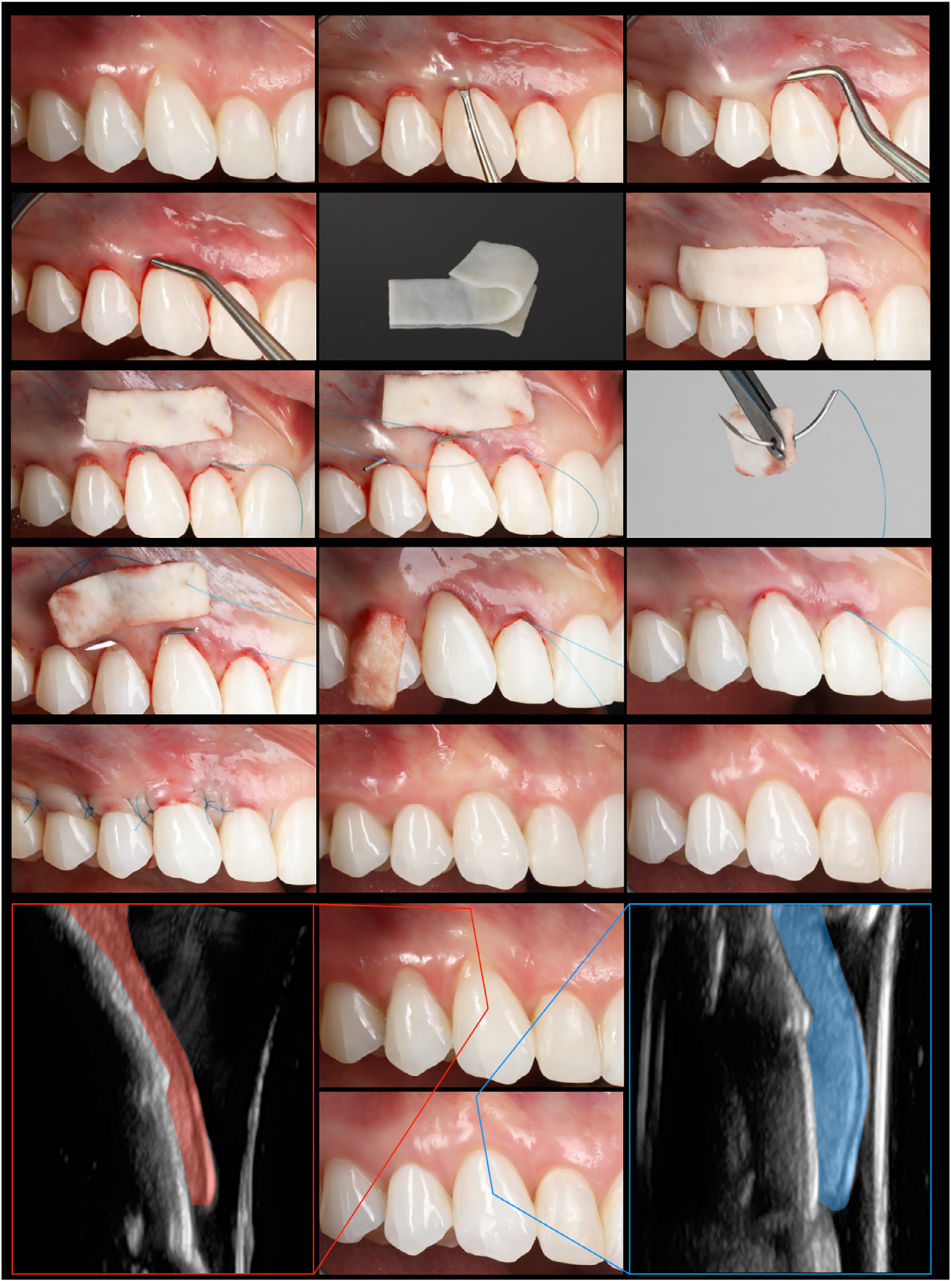
Tunnel technique in combination with a human‐derived acellular dermal matrix (AlloDerm Select, BioHorizons, USA) for the treatment of multiple recessions in the right maxilla. The healing at 1 year and 3 years are reported. The ultrasound scans display in red the soft tissue of the canine prior to the root coverage procedure, and in blue the same anatomical region at the 3 year‐follow‐up. Reproduced with permission from John Wiley and Sons.^207^

**FIGURE 7:**
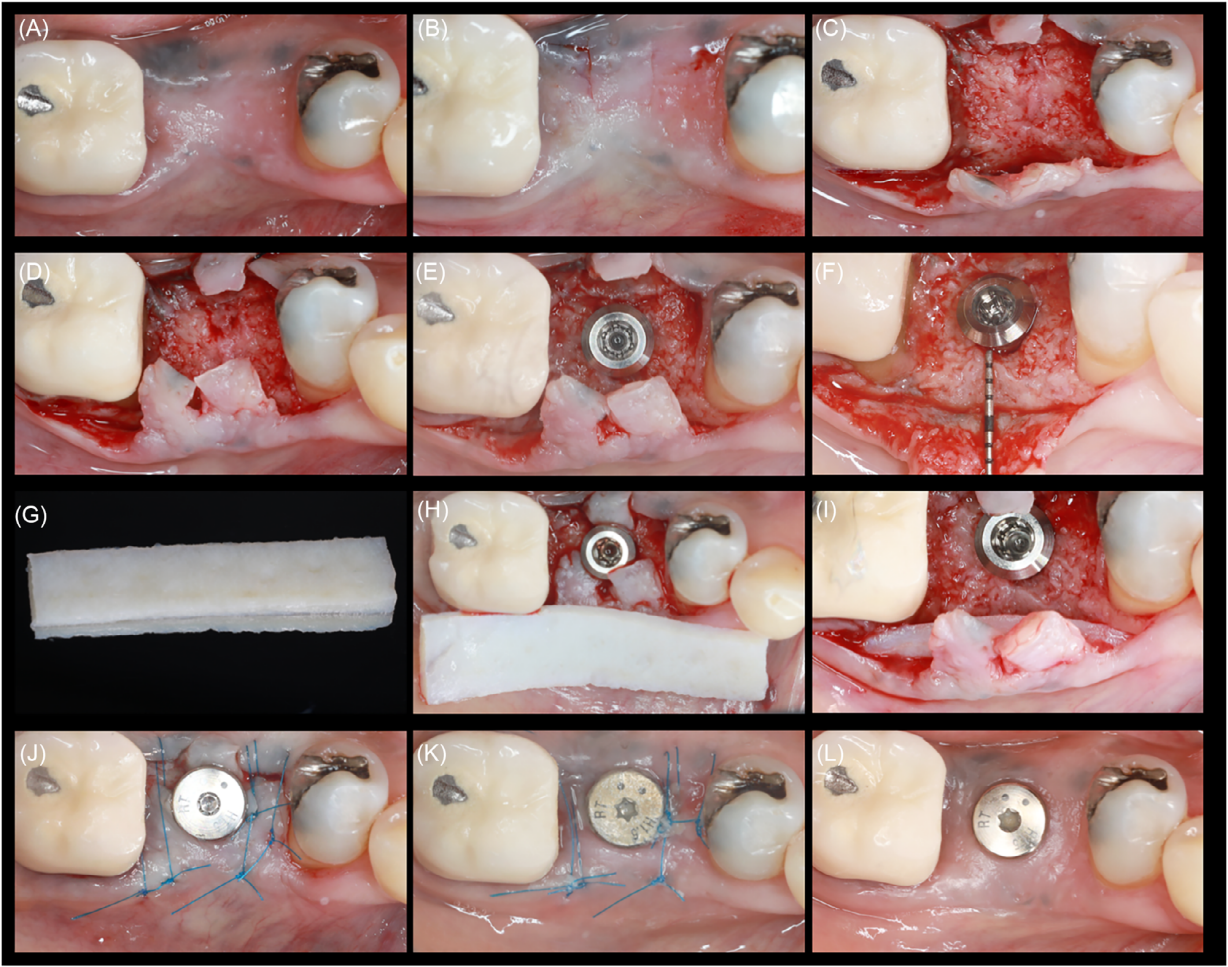
Horizontal soft tissue augmentation at the time of implant placement. After an incision design aimed at moving some keratinized mucosa from the lingual to the buccal aspect, the flap was raised, and an implant was placed (A–E). The site had an adequate buccal bone and keratinized mucosa width, while the mucosal thickness at the implant site, as well as the gingival thickness at the adjacent dentition, were limited (F). A porcine‐derived acellular dermal matrix (pADM, NovoMatrix, BioHorizons Camlog Italia, Italy) was trimmed according to the recipient site and was secured with the flap to recreate an adequate ridge contour and to increase soft tissue thickness around the implant and the adjacent teeth (G–J). Healing at 3 weeks (K) and 4 months (L). Reproduced with permission from John Wiley and Sons.^207^

**FIGURE 8:**
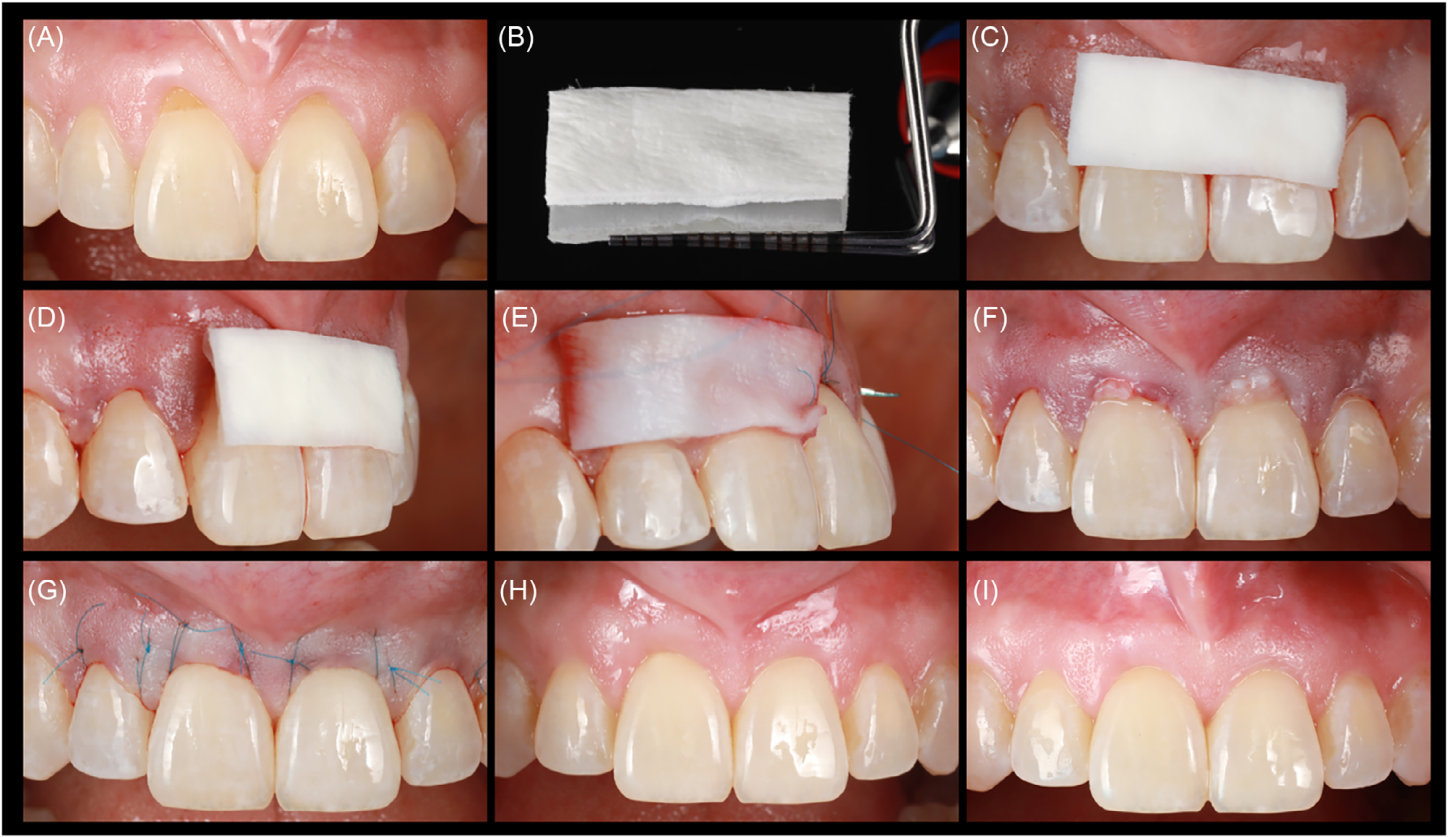
Tunnel technique in combination with a xenogeneic collagen matrix (Geistlich Mucograft, Geistlich Pharma, Switzerland) for the treatment of gingival recessions on the two maxillary central incisors. (A) Baseline. (B) Xenogeneic collagen matrix. (C,D) Graft in position after the preparation of the tunnel flap. (E,F) Insertion of the graft underneath the tunnel flap. (G) Suturing. (H) Outcome at 6 months. (I) Outcome at 3 years. Reproduced with permission from John Wiley and Sons.^207^

Scaffolds in oral soft tissue regenerative procedures each have distinct biological and structural features that contribute to their clinical performance. Human acellular dermal matrix (hADM), derived from cadaveric human skin and processed to retain the extracellular matrix while eliminating immunogenic components, has long been used in medicine and was the first substitute applied in dentistry for soft tissue augmentation.^186^, ^187^, ^188^ hADM serves as a biocompatible scaffold that promotes revascularization and host cell migration but requires submerged healing to avoid sloughing or necrosis^102^, ^186^, ^189^, ^190^, ^191^, ^192^, ^193^, ^194^ (Figure 6). Similarly, porcine‐derived acellular dermal matrices (pADM) act as three‐dimensional collagenous scaffolds that support fibroblast and endothelial cell proliferation, contributing to rapid vascularization and integration^195^, ^196^, ^197^ (Figure 7). Xenogeneic collagen matrices offer additional alternatives. The first‐generation bilayered collagen matrix (CMX) features one compact, occlusive layer to provide stability and one porous layer that encourages clot stabilization, angiogenesis, and cellular ingrowth.^198^, ^199^ CMX can heal by secondary intention and has been used successfully in both tooth‐ and implant‐related applications, including keratinized tissue augmentation and ridge preservation.^112^, ^200^, ^201^ Histological studies confirm its biocompatibility and complete integration without eliciting adverse inflammatory responses^198^, ^202^, ^203^, ^204^, ^205^ (Figure 8). The volume‐stable collagen matrix (VCMX), a second‐generation scaffold, incorporates cross‐linked collagen in a porous, trabecular structure that supports cell migration and angiogenesis, while resisting early degradation.^32^, ^33^, ^34^, ^35^ Preclinical studies have demonstrated that VCMX maintains volumetric integrity during early healing and facilitates rapid cellular infiltration and neovascularization. After 90 days, most of the VCMX is typically replaced with newly formed connective tissue.^206^

The CTG should be preferred at sites lacking/limited keratinized tissue, as it appears to uniquely promote consistent keratinization of the alveolar mucosa when applied in bilaminar procedures.^22^, ^177^ It may also be preferred in deep or complex gingival recessions where maximum tissue augmentation is required.^208^, ^209^, ^210^, ^211^ In less complex scenarios, however—especially where patient comfort and minimally invasive approaches are prioritized—graft substitutes offer a highly viable alternative.^38^, ^169^, ^173^

### 4.3 Peri‐implant soft tissue phenotype modification

Importantly, all grafting modalities demonstrated superior performance in increasing GT compared with flap procedures alone. This has significant implications for long‐term root coverage stability. Studies have shown that sites treated with flap procedures alone are more prone to recession relapse,^1^, ^212^, ^213^, ^214^ while bilaminar techniques—with or without substitutes—are associated with better stability, likely due to a more durable soft tissue phenotype.^1^, ^29^, ^213^, ^215^, ^216^, ^217^ Recently, in a long‐term follow‐up study of previous RCTs, we found that a GT ≥ 1.46 mm and a KT width ≥ 1.5 mm at 6 months following the treatment of gingival recessions were the two prominent factors predicting stability of the gingival margin over 10 years.^2^

Similarly, in the context of implant therapy, all evaluated interventions led to significant increases in peri‐implant mucosal thickness (MT), with CTG once again demonstrating the highest outcomes. The efficacy of graft substitutes at both tooth and implant sites can be explained by their structural properties. These biomaterials, though acellular, provide a scaffold that supports host cell migration, vascularization, and integration.^22^ MT augmentation was also associated with a significant reduction in probing depth (PD), a finding that aligns with the concept of a “soft tissue seal” at implant sites. This seal—particularly when composed of thick, adherent keratinized mucosa—may protect against plaque accumulation and peri‐implant disease.^218^, ^219^ Accordingly, the modification of peri‐implant soft tissue phenotype has been associated with improved long‐term outcomes and a reduced complication rate.^49^, ^220^, ^221^, ^222^

### 4.4 Surgical innovations and flap design

The findings of this review should be interpreted within the broader trajectory of surgical innovation in soft tissue grafting. Successive refinements from CAF to tunneling techniques, and more recently TCAF, illustrate how advances in flap design have paralleled the shift toward minimally invasive, site‐specific strategies. These developments highlight that improved regenerative outcomes are not solely dependent on grafting materials or biologics, but also on the surgical platforms that enable their effective use.^4^, ^136^, ^138^ As such, graft selection in implant therapy should also be personalized, taking into account anatomical constraints, esthetic requirements, and the patient's tolerance for surgical complexity.^223^ The shift toward biologically driven, patient‐specific regenerative strategies calls for flexible protocols that integrate both clinical efficacy and patient experience. In this context, contemporary soft tissue augmentation should ideally adhere to the following principles:

1. Minimally invasive flap designs.
2. Selective and conservative use of CTG, when maximum efficacy is critical.
3. Use of graft substitutes or biomaterials alone or in combination with autogenous tissue, based on case‐specific factors.^30^, ^169^, ^170^, ^174^, ^224^, ^225^

### 4.5 Emerging strategies in hard tissue regeneration

**FIGURE 9:**
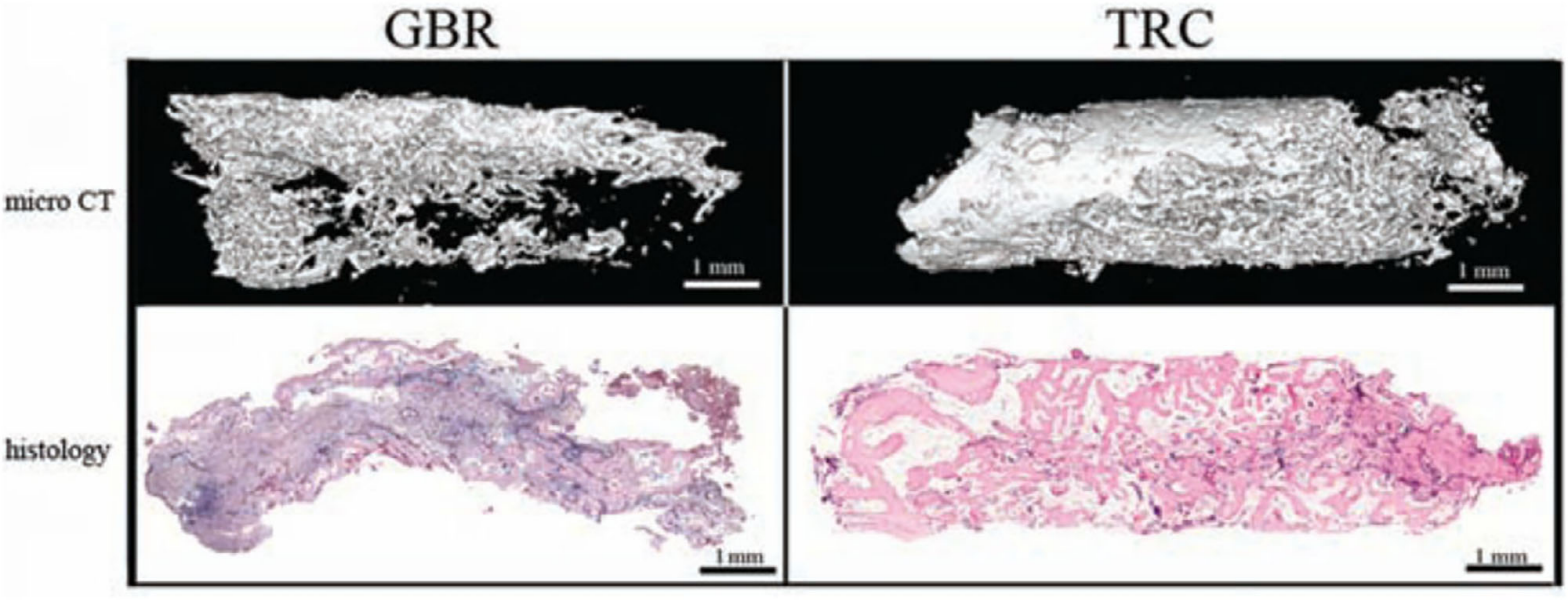
Tissue repair cells (TRCs) promote regeneration of alveolar bone defects. Micro‐computed tomographic (CT) and histomorphometric analyses (H&E staining) of bone formation in a representative specimen from a GBR‐treated site and a TRC treated site 6 weeks following treatment (original magnification: 2×). Both sections show varying degrees of mature cortical bone with high vascularity, as indicated by the abundance of blood vessels. Bone volume fraction (BVF), bone mineral density (BMD), and histomorphometric measures were quantified and compared between TRC and GBR treated sites at 6 and 12 weeks. (Reproduced with permission from Kaigler et al.^152^).

In the context of emerging approaches for periodontal and alveolar bone regeneration, there are limited controlled, prospective clinical investigations of these approaches, which include stem cell therapy, 3D bioprinting, and “smart” materials. Despite the limited number of clinical studies to date, clinicians seek to learn more about these approaches and how they can be incorporated into their practice.^226^ The proof‐of‐concept studies which have been conducted overall demonstrate accelerated and enhanced abundance and volume of regenerated bone particularly in the context of stem cell therapy. Additionally, the quality of regenerated bone is a crucial component in evaluating the success of regenerative therapies and in those studies evaluating histomorphometric outcomes these parameters have been observed to be enhanced^152^ (Figure 9). As clearly presented in medicine, and many of the included studies in our field,^143^, ^144^, ^227^, ^228^, ^229^ the regenerated bone following application of stem cells exhibits enhanced vascularity, bone trabeculation, and enhanced turnover of the biomaterial carrier, all which play critical roles in the long‐term success of any regenerative approach.

Despite CAD‐CAM technologies being used in dentistry for many years now, until recently, they have been used primarily in the laboratory and prosthetic realms of dentistry. The increasing awareness and emerging exploration of their potential in surgical reconstruction for hard tissue regeneration is gaining traction and widespread attention. Although these approaches undoubtedly require additional resources, armamentarium, and increased preoperative planning time, the early clinical investigations of their utility appear to have benefits of decreased surgical time, decreased patient morbidity, and improved patient satisfaction.^168^ Future investigations are warranted to determine the true feasibility, patient benefit, and cost effectiveness of these approaches for routine, day–day use.^168^ If clinical outcomes and patient benefit are demonstrated to be superior with these approaches, they will at minimum be utilized for larger reconstructive regenerative applications regardless of what the cost of these treatments turns out to be (additional limitations are discussed in the Supplementary Appendix).

### 4.6 AI (artificial intelligence) in regeneration

Lastly, it should be mentioned that while the aforementioned emerging technologies have shown early promising results in preclinical studies and clinical trials, challenges persist in identifying the most appropriate cases to treat, the ideal treatment modality to use, defining desired outcome measures, and establishing predictability for efficacious outcomes. It is in this realm that there is the opportunity for AI to play a role in personalized regenerative therapies by enabling the ability to synthesize large datasets to better understand how the multifaceted variables of different treatment modalities impact and yield their respective outcomes.^15^, ^230^, ^231^ Artificial intelligence is broadly defined by the ability and utilization of computer systems to perform tasks that would normally require human intelligence including learning, reasoning, perceiving, and problem‐solving.^12^ Despite the concept of AI being around for decades, it has only been recently that the plausibility of its use in regenerative medicine and dentistry has been considered due to advances in machine learning, deep learning, and molecular and biomedical tools for generating large and complex biological datasets.^230^ Through synthesis of large datasets across a broad range of studies relative to patient specific factors (i.e., age, sex, medical status), site specific factors (i.e., defect etiology, defect size), treatment modalities, and outcomes, AI algorithms can be developed to predict which regenerative approach will be most effective in treating a specific clinical scenario. In the context of stem cell therapies and scaffold technologies for personalized hard and soft tissue regenerative applications, AI can identify the best sourcing and conditions for cultivation of cells, determine optimal dosing, and determine optimum design parameters for scaffolds.^232^, ^233^ Over the next 10 years, AI will play a significant role in not only our ability to personalize treatment planning for regenerative approaches, but also our ability to personalize the surgical approaches and supportive follow‐up care regimens for patients undergoing these therapies.

## 5 CONCLUSIONS

Based on the current available evidence and the inherent limitations of this review, the following conclusions can be drawn:

All soft tissue grafting modalities, including CTG, hADM, pADM, CMX, and VCMX, significantly increase soft tissue thickness (GT and MT) compared with flap procedures alone.

CTG remains the graft associated with the greatest gain in GT and MT. While it showed the highest estimates in the statistical models, no statistically significant differences were observed between CTG and the grafting materials hADM, pADM, and VCMX. These analyses support the clinical utility of soft tissue substitutes, especially when patient comfort and surgical morbidity are prioritized.

The selection of the grafting biomaterial should be based on anatomical considerations, clinical objectives, and patient‐specific factors, including esthetic demands and expectations.

Future applications of these substitutes may extend into other regenerative procedures, including tissue conditioning prior to implant placement, immediate implant protocols, or minimally invasive treatment of peri‐implant soft tissue deficiencies.

Stem cell therapies for bone regeneration have demonstrated safety and efficacy in early clinical investigations for various defect types including those that result in tooth‐loss associated atrophy, sinus pneumatization, trauma, and congenital cleft defects.

The merging of image‐based, personalized technologies with stem cell therapy has the potential for more targeted treatment approaches to optimize clinical outcomes and patient satisfaction while minimizing surgical time and post‐operative complications.

For bone regeneration the parameters of cost effectiveness, clinical efficacy, patient satisfaction, and clinical acceptance will ultimately determine to what extent emerging technologies will be adopted into clinical practice.

The emergence of cell‐free approaches to hard tissue regeneration has the potential to help in overcoming regulatory challenges associated with cell therapy and further, the incorporation of artificial intelligence in these approaches can make them more personalized to enhance predictability of outcomes.

The application of personalized and novel biomaterial constructs are at an early stage of development for soft and hard tissue regeneration around teeth and dental implants. More study is required to take preclinical findings to human clinical investigations.

## AUTHOR CONTRIBUTIONS

**Shayan Barootchi**: Designed the study; performed the literature search, initial screening, and article selection; extracted the data and assessed the risk of bias; conducted the statistical analysis and led the writing. **Lorenzo Tavelli**: Designed the study; extracted the data and assessed the risk of bias; conducted the statistical analysis and led the writing. **Hamoun Sabri**: Designed the study; performed the literature search, initial screening, and article selection; extracted the data and assessed the risk of bias. **William V. Giannobile**: Contributed to the study methodology; aided in drafting and critically revising the manuscript. **Darnell Kaigler**: Contributed to the study methodology; aided in drafting and critically revising the manuscript.

## CONFLICT OF INTEREST STATEMENT

Drs. Shayan Barootchi and Lorenzo Tavelli have provided lectures sponsored by Geistlich Pharma AG, Wolhusen, Switzerland and Lynch Biologics, Franklin, Tennessee, USA.

## Supporting information



Supporting Information
