# Supplementary material for: Emerging, personalized, and minimally invasive technologies for periodontal and peri‐implant soft and hard tissue regenerative therapy
Source: J Periodontol. 2026 Sep 14;97(Suppl 1):S103–39. doi: 10.1002/jper.70108 (PMC13575464; doi:10.1002/jper.70108)
Supplement: Supplementary file 1 [file JPER-97-S103-s001.docx]

**Supplementary Appendix**

**Emerging, personalized, and minimally-invasive technologies for periodontal and peri-implant soft and hard tissue regenerative therapy**

Shayan Barootchi, Lorenzo Tavelli, Hamoun Sabri, William Giannobile, Darnell Kaigler

**Page 2**	The set inclusion and exclusion criteria

**Page 4**	The electronic and manual search strategy

**Page 7**	Study selection and data extraction

**Page 9**	Criteria for assessment of the risk of bias for studies on soft tissue regeneration around teeth and implants

**Page 10**	Grading the certainty of evidence for quantitative assessment on soft tissue regeneration

**Page 11**	Detailed results on the literature search

**Page 14**	Characteristics of the included research and qualitative results on soft tissue regeneration around teeth and implants

**Page 15**	Quality assessment and “Risk of bias” report – teeth

**Page 16**	Quality assessment and “Risk of bias” report – implants

**Page 17**	Discussion – limitations

**Page 18**	Online references

**The set inclusion and exclusion criteria for directing the systematic literature search are as follows:**

**Studies focusing on defects around teeth need to have adhered to the following criteria:**

**Inclusion Criteria:**

- Randomized clinical trials (RCTs)
- Duration of follow-up reaching at least 12 months for root coverage (without limitations for the final follow-up).
- Studies reporting on the outcomes of periodontal plastic procedures, whether for the treatment of gingival recession defects, or for increase in the amount of gingival thickness (GT)
- Studies that provide data on at the outcome measure of GT

**Exclusion:**

- Non-randomized studies (non-randomized prospective studies, case control studies, case series, case reports)
- Studies with less than 12 months of follow-up for root coverage
- Studies that dd not provide details on the components of the periodontal soft tissue phenotype.
- Studies with a sample size of less than 10 patients
- Combination therapies (e.g., simultaneous mixture of multiple soft tissue grafts, or grafts with biologics/healing enhancers) if individual details were not provided on the outcomes or treatments
- Laser treatment as the method of regenerative protocol
- Studies investigating solely non-carious cervical lesions
- Studies investigating a specific population cohort (such as studies on smoking individuals, etc.)

**Studies focusing on defects around implants need to have adhered to the following criteria:**

**Inclusion Criteria**

- Soft tissue augmentation at implant sites using a free gingival graft, a connective tissue graft, an acellular dermal matrix, or a collagen matrix
- Prospectively conducted human interventional RCTs
- Evaluation and reporting of clinical outcomes of interest (keratinized mucosa width, mucosal thickness or soft tissue height) over a minimum follow-up period of 6 months.

**Exclusion Criteria**

- Retrospective clinical studies, case reports or animal studies
- A final follow-up of below 6 months
- Inclusion of implants with a diagnosis of peri-implantitis ^1^
- Simultaneous hard and soft tissue augmentation. For the quantitative analysis any treatment arm that included bone augmentation was excluded from the analysis.
- Studies investigating a specific population cohort (such as studies on smoking individuals, etc.)

**Information regarding the search strategy:**

As per the stated focused questions, two separate search strategies were performed. The initial search strategy was for augmentation around teeth, followed by a search strategy for augmentation around implants.

The search was initiated with a computerized systematic search in the following electronic data bases for selection of trials that met the aformentioned inclusion criteria: The national library of Medicine (MEDLINE via PubMed); EMBASE via OVID; the Cochrane Central Register of Controlled Trials; and Latin American & Caribbean Health Sciences Literature (LILACS), Web of Science, and Scopus.

To search for unpublished trials, grey literature, nonprofit reports, government research or other materials, ClinicalTrial.gov and OpenGrey ([www.opengrey.eu](http://www.opengrey.eu)) were also explored.

The search strategy was first designed using a string of medical subject headings and free text terms for the MEDLINE database and then modified appropriately to be used in other database searches.

The utilized electronic search keywords for studies on natural dentition are as follows:

**MEDLINE:**

((((gingival recession) OR root coverage) OR periodontal plastic surgery) AND human) AND randomized clinical trial

(((("gingival recession"[MeSH Terms] OR ("gingival"[All Fields] AND "recession"[All Fields]) OR "gingival recession"[All Fields]) OR "root"[All Fields]) AND ("AHIP Cover"[Journal] OR "coverage"[All Fields]))) OR (periodontal[All Fields] AND ("surgery, plastic"[MeSH Terms] OR ("surgery"[All Fields] AND "plastic"[All Fields]) OR "plastic surgery"[All Fields] OR ("plastic"[All Fields] AND "surgery"[All Fields])))) AND ("humans"[MeSH Terms] OR "humans"[All Fields] OR "human"[All Fields])) AND ("randomized controlled trial"[Publication Type] OR "randomized controlled trials as topic"[MeSH Terms] OR "randomized clinical trial"[All Fields] OR "randomised clinical trial"[All Fields])

**EMBASE:**

('gingival recession'/exp *OR* 'gingival recession' *OR* (gingival AND recession) *OR* 'root coverage' *OR* (('root'/exp OR root) *AND* coverage) *OR* 'periodontal plastic surgery' *OR* (periodontal *AND* ('plastic'/exp *OR* plastic) *AND* ('surgery'/exp OR surgery))) *AND* ('human'/exp *OR* human) *AND* 'randomized clinical trial'

**Cochrane:**

“Gingival Recession” [Search All Text] *OR* “Root Coverage” [Search All Text] *OR* “Periodontal Plastic Surgery” [Search All Text].

**LILACS:**

(tw:(Gingival Recession)) *OR* (tw:(Root Coverage)) *OR* (tw:(Periodontal Plastic Surgery)) *AND* (tw:(randomized clinical trial)) *OR* (tw:(randomised clinical trial))

**Scopus:**

( TITLE-ABS-KEY ( gingival AND recession OR ( "gingival" [all AND fields] AND "recession" [all AND fields] ) ) OR TITLE-ABS-KEY ( root AND coverage ) OR TITLE-ABS-KEY ( gingival AND augmentation ) OR TITLE-ABS-KEY ( periodontal AND plastic AND procedure ) OR TITLE-ABS-KEY ( periodontal AND plastic AND surgery ) AND TITLE-ABS-KEY ( human ) AND TITLE-ABS-KEY ( randomised AND clinical AND trial ) OR TITLE-ABS-KEY ( randomised AND clinical AND trial ) )

**Web of Science:**

**ALL FIELDS:** (gingival recession) *OR* **ALL FIELDS:** (gingival augmentation) *OR* **ALL FIELDS:** (periodontal plastic surgery) *OR* **ALL FIELDS:** (periodontal plastic procedures) *OR* **ALL FIELDS:** (periodontal phenotype) *AND* **ALL FIELDS:** (gingival phenotype) *AND* **ALL FIELDS:** (human) *OR* **ALL FIELDS:** (randomized clinical trial) *AND* **TOPIC:** (randomised clinical trial)

**Refined by:** [excluding] **DOCUMENT TYPES:** ( REVIEW ) AND **WEB OF SCIENCE CATEGORIES:** ( DENTISTRY ORAL SURGERY MEDICINE )

**OpenGrey:**

(gingival *OR* gingival recession *OR* root coverage *OR* periodontal plastic procedure)

**ClinicalTrials.gov:**

Completed Studies | Gingival Recession OR root coverage *OR* periodontal plastic procedure

The utilized electronic search keywords for studies on dental implants are as follows:

**MEDLINE:**

(((((((("acellular dermal matrix"[All Fields] OR "dermal matrix allograft"[All Fields]) OR "collagen matrix"[All Fields]) OR "connective tissue graft"[All Fields]) OR "free gingival graft"[All Fields]) OR "vestibuloplasty"[All Fields]) OR "soft tissue augmentation"[All Fields]) OR "apically positioned flap"[All Fields]) AND "dental implants"[All Fields]) OR "jaw, edentulous"[All Fields] AND (Clinical Study[ptyp] AND "humans"[MeSH Terms] AND jsubsetd[text])

**EMBASE:**

('dental implants' AND 'acellular dermal matrix' OR 'dermal matrix allograft' OR 'collagen matrix':af OR 'connective tissue graft' OR 'free gingival graft' OR 'vestibuloplasty'/exp OR 'vestibuloplasty' OR 'soft tissue augmentation'/exp OR 'soft tissue augmentation' OR 'apically positioned flap') AND 'article' AND 'clinical'

**Cochrane:**

("Dental implants" OR "edentulous jaw") AND ("acellular dermal matrix" OR "collagen matrix" OR "connective tissue graft" OR "free gingival graft")

**LILACS:**

(tw:(acellular dermal matrix)) OR (tw:(dermal matrix allograft)) OR (tw:(collagen matrix)) OR (tw:(connective tissue graft)) OR (tw:(free gingival graft)) OR (tw:(free soft tissue graft)) OR (tw:(apically positioned flap)) AND (tw:(dental implants))

**Scopus:**

( TITLE-ABS-KEY ( acellular AND dermal AND matrix ) OR TITLE-ABS-KEY ( dermal AND matrix AND allograft ) OR TITLE-ABS-KEY ( collagen AND matrix ) OR TITLE-ABS-KEY ( connective AND tissue AND graft ) OR TITLE-ABS-KEY ( free AND gingival AND graft ) OR TITLE-ABS-KEY ( soft AND tissue AND augmentation ) OR TITLE-ABS-KEY ( apically AND positioned AND flap ) AND TITLE-ABS-KEY ( dental AND implants ) AND NOT TITLE-ABS-KEY ( animal ) )

**Web of Science:**

ALL FIELDS: (acellular dermal matrix) OR ALL FIELDS: (dermal matrix allograft) OR ALL FIELDS: (collagen matrix) OR ALL FIELDS: (connective tissue graft) OR ALL FIELDS: (free gingival graft) OR ALL FIELDS: (soft tissue augmentation) OR ALL FIELDS: (apically positioned flap) AND ALL FIELDS: (dental implants) NOT TITLE: (Animal)

Refined by: [excluding] DOCUMENT TYPES: ( EDITORIAL MATERIAL OR RETRACTED PUBLICATION OR REVIEW OR LETTER OR PROCEEDINGS PAPER OR MEETING ABSTRACT OR NOTE ) AND WEB OF SCIENCE CATEGORIES: ( ENGINEERING BIOMEDICAL OR DENTISTRY ORAL SURGERY MEDICINE OR MEDICINE RESEARCH EXPERIMENTAL )

Timespan: All years. Indexes: SCI-EXPANDED, SSCI, A&HCI, CPCI-S, CPCI-SSH, BKCI-S, BKCI-SSH, ESCI, CCR-EXPANDED

**OpenGrey:**

dental implants AND connective tissue graft OR dermal matrix allograft OR collagen matrix OR free gingival graft OR free soft tissue graft OR apically positioned flap

**ClinicalTrials.gov:**

Studies With Results | dental implants AND connective tissue graft OR dermal matrix allograft OR collagen matrix OR free gingival graft OR free soft tissue graft OR apically positioned flap

No restrictions were assigned regarding the date of publication, journal, or the language used. Next, the search results were downloaded to a bibliographic database to facilitate duplicate removal and cross-reference checks. The last search was conducted on January 1, 2025.

In addition, to ensure a thorough screening process, the electronic search was complimented by an additional manual search of the following journals: *Journal of Dental Research, Journal of Clinical Periodontology, Journal of Periodontology, Clinical Oral Investigations, and International Journal of Periodontics* and *Restorative Dentistry*. The manual search in the referenced sources was performed from January 1, 2000 to February 1, 2025. The reference lists of the retrieved studies for full-text screening and previous reviews investigating periodontal plastic procedures were also screened for missing records and article identification.

**Study selection and data extraction:**

Two pre-calibrated review authors (SB, HS) the titles and abstracts (as available) of the studies identified in the searches, in duplicate and independently. Subsequently, the full text of all publications appearing to meet the inclusion criteria or for which there was not sufficient information in the title and abstract to make a decision, were obtained.

At this first stage, any study considered as potentially relevant by at least one of the reviewers was included for the next screening phase. Subsequently, the full-text publications were also evaluated in duplicate and independently by the same examiners. The examiners were calibrated with the first 10 full-text, consecutive publications. Any disagreement on the eligibility of studies was resolved through discussion between both reviewers until consensus was reached or through arbitration by a third reviewer (LT). All potentially relevant studies that did not meet the eligibility criteria were excluded and the reasons for exclusion was recorded. Inter-examiner agreement following full-text assessment was calculated via kappa statistics.

In the case of multiple retrieved publications reporting on the same study or investigating the same population at different follow-up intervals and/or secondary analysis of the same data, a decision was made to pool together all relevant data as a single comprehensive report with the most complete data for inclusion in the analysis. SB and HS assessed the pooled studies independently and included only those reporting data on the primary outcome (soft tissue augmentation around teeth (GT), and implants (MT)) assessed in this review for the original study sample. Disagreement on the selection of the studies was resolved in the same manner as previously mentioned.

Data management was performed by two review authors (SB, HS). This was done independently by extracting all relevant details from the selected trials using a predetermined data extraction form specifically designed for this review which was first piloted in a small number of studies. At each stage, any debate between the reviewers was resolved through discussion and consensus. If a disagreement persisted, the judgment of a third reviewer (LT) was decisive. Aside from the primary outcome, the following study details were extracted:

- Type of study, number of centers, geographic location, sample frame (university vs. private practice), source of funding
- Population characteristics, age of participants, number of participants and treated sites (baseline/follow-up), singular/multiple treated sites, duration of follow-up
- Type of intervention, utilization of a graft material, the type of graft, techniques
- Clinical measurements relating to the treated site(s) at baseline and at each respective follow-up recall, and their method of measurement. All values were extracted from the selected publications (their means ± standard deviations (SD)).

When dealt with missing data, if a study did not provide any information on the primary outcomes (GT and MT), attempts were made to contact the authors of that study to obtain the missing values. If the attempts were not successful, the trial was excluded.

**Criteria for assessment of the risk of bias for the included randomized clinical trials for the outcome of soft tissue regeneration around teeth and dental implants**

The Cochrane Risk of Bias 2 (RoB-2)^2^ was used to assess the risk of bias of the included RCTs. This tool is specifically designed for critically evaluating the internal validity of RCTs within the context of systematic reviews.

RoB-2 provides a meticulous framework for assessing bias across five key domains:

**1. Randomization Process:** This domain delves into the methods employed to generate and maintain randomness in participant allocation. It seeks to ensure that interventions are assigned unpredictably, eliminating systematic bias and guaranteeing equal probability of participation in each treatment group.

**2. Allocation Concealment:** This domain focuses on masking the treatment allocation sequence until participants are enrolled. By preventing anticipation of assigned interventions, it guards against selection bias, which can skew sample characteristics and undermine study validity.

**3. Blinding of Participants and Personnel:** This domain aims to minimize performance bias by keeping both participants and study personnel (researchers, healthcare providers) unaware of their assigned intervention groups. This prevents unintentional modifications in behavior or data collection influenced by knowledge of group allocation.

**4. Blinding of Outcome Assessment:** Similar to the previous domain, this focuses on blinding outcome assessors. By safeguarding against detection bias, it ensures objective and unbiased evaluation of intervention effects, regardless of group knowledge.

**5. Incomplete Outcome Data:** This domain scrutinizes the management of missing data and participant dropouts. It requires justification for missingness to rule out bias in data loss and emphasizes the use of appropriate methods like intention-to-treat analysis to maintain the integrity of analysis and conclusions.

Each domain is evaluated separately for every outcome within the study. The authors answered one or more signaling questions within each domain. The available response options were “yes,” “probably yes,” “probably no,” “no,” and “no information.” These answers able judgments like “low risk of bias,” “some concerns,” or “high risk of bias”. An overall risk of bias judgment is obtained across the judgments in each domain.

**Grading the certainty of evidence for quantitative assessment on soft tissue regeneration**

For the quantitative assessment on soft tissue regeneration around teeth and implants, we employed a modified approach to the Grading of Recommendations Assessment, Development, and Evaluation (GRADE) framework^3^, as described in a recent report to evaluate the certainty of evidence. Given the nature of our analysis, which utilized a mixed-model-based regression network meta-analysis (NMA) rather than traditional pairwise meta-analyses or conventional networks, we adapted our modification to suit this context. Our assessment focused on the following key domains:

1. Estimate and its 95% confidence intervals (CIs), alongside associated p-values.

2. Study design, considering the rigor and appropriateness of the included studies.

3. Population characteristics, including sample size and the number of implants, to gauge the representativeness of the study populations.

4. Follow-up duration, assessing the adequacy of the observational period for capturing relevant outcomes.

5. Risk of bias (ROB), evaluating the methodological quality of the included studies

6. Inconsistency, examining the agreement or consistency among study findings.

7. Indirectness, assessing the relevance and applicability of the evidence to the research question.

8. Imprecision, considering the precision of effect estimates, particularly in relation to confidence intervals and sample size.

9. Other relevant considerations specific to our study context

In culmination, the overall certainty of evidence with regards to each main outcome of the study, across these domains was assessed to be categorized as Very Low, Low, Moderate, or High.

**Search results and study selection for studies on soft tissue regeneration aroud teeth and dental implants:**

The electronic and manual search was conducted in two phases: one focusing on studies related to natural dentition, and the other on augmentation around dental implants. Figure 1 outlines this process.

**Figure 1.** The computerized systematic and manual literature search process and the screening of the articles for identifying the included studies in this review. The top flowchart shows the process of arriving at the included articles around teeth, and the bottom flowchart displays the process for articles around implants. * refers to search in the grey literature.

The search for studies on natural dentition yielded a total of 5,253 articles from the electronic databases. 2,938 inputs were then excluded after duplicate removal, leading to a total of 2,793 records that were reviewed individually by both authors via title and abstract screening. As a result, 99 records remained and were subjected to full-text assessment. After further exclusion of articles, as per the eligibility criteria, 54 publications remained for inclusion in the analysis. Table 1 of this Appendix provides the references and the reasons for the excluded articles at this stage.

The electronic search for dental implants resulted in 7,565 articles. After duplicate removal, 2,876 studies remained and were evaluated by reviewing titles and abstracts, which led to an eventual full-text assessment of 69 articles by both reviewers. After further screening according to the inclusion criteria, 24 publications remained and were included in the final analysis. Table 2 of this Appendix provides the references and the reasons for the excluded articles.

Inter-examiner agreement during both the initial screening and full-text inclusion phases was excellent (kappa scores of 0.89 and 0.88 for initial screening for studies on teeth, and implants, and 0.93 and 0.91 for full-text assessment for studies on teeth and implants, respectively)^4^.

**Supplementary Table 1:** Reference and the reason for the excluded articles for search on natural dentition.

| **References** | **Rationale** |
| --- | --- |
| Berlucchi et al. 2005, Cordioli et al. 2001, Francetti et al. 2018, Hirsch et al. 2005, Moses et al. 2006, Nemcovsky et al. 2004, Pini Prato et al. 1996, Pini Prato et al. 1992, Pini Prato et al. 2010 ^5-13^ | Non-randomized clinical trial |
| Ricci et al. 1996 ^14^, Bakhishov et al. 2021, Cesar Neto et al. 2024, Louis et al. 2023 | Incomplete published dataset |
| Carney et al. 2012, Gilbert et al. 2015, Gupta et al. 2018, Harris 1997, Harris 2000, Harris 2001, Harris 2005, Joly et al. 2007, Keceli et al. 2015, Kimble et al. 2004, Lafzi et al. 2016, Muller et al. 1999, Pini Prato et al. 1999, Tatakis and Trombelli 2000, Tonetti et al. 2018, Woodyard et al. 2004, Zucchelli et al. 2009 ^15-31^ | Below 12 months follow-up |
| Aydinyurt et al. 2019 ^32^, Carnio et al. 2015, McGuire et al. 2005, 2008, 2011, 2014, Dorfman et al. 1980, Kennedy et al. 1985, Carcuac et al. 2022 | No data on gingival thickness |
| Stähli et al. 2022, Skierska et al. 2024, Louis et al. 2023 | Mixture of biologics/bioactive mediators |
| Peter et al. 2023 | Below required sample size for the interventional trial |
| Costa et al. 2016, Reino et al. 2012 ^33, 34^ | Inclusion criteria: smoking patients |

**Supplementary Table 2:** Reference and the reason for the excluded articles for search on dental implants.

| **References** | **Rationale** |
| --- | --- |
| Baltacioglu et al. 2016, Migliorati et al. 2015, Park and Wang 2007, Park et al. 2008, Sanz-Martin et al. 2019, van Nimwegen et al. 2018, Yoshino et al. 2014, Zuiderveld et al. 2018 ^35-42^, Al-Tarawneh et al. 2024 | Simultaneous bone + soft tissue augmentation (9) |
| Almeida et al. 2009, Boynuegri et al. 2013, Cornelini et al. 2008, Hanser & Khoury 2016, Roccuzzo et al. 2014, Roccuzzo et al. 2019, Schneider et al. 2011 ^43-49^ | Lacking or with incomplete information  (7) |
| Oh et al. 2017 ^50^, Basegmez et al. 2012 ^51^, Vellis et al. 2019 ^52^, Buyukozdemir Askin et al. 2015 ^53^, Lorenzo et al. 2012 ^54^, Basegmez et al. 2013 ^55^, Farooqui et al. 2023, Ramanauskaite et al. 2023, Solderer et al. 2024, Puisys et al. 2024, Puisys et al. 2022 | Lack of data on mucosal thickness  (9) |
| Anderson et al. 2014 ^56^, Cairo et al. 2017 ^57^, Froum et al. 2015 ^58^, Puzio et al. 2018 ^59^, Ustaoglu et al. 2020 ^60^, Hutton et al. 2018 ^61^, Hämmerle et al. 2023, Papapetros et al. 2019, Ashurko et al. 2022, Thoma et al. 2016; Zeltner et al. 2017; Rojo et al. 2018, | Below 6 months data  (12) |
| Akcali et al. 2015, Artiushkevich et al. 1990, Barone et al. 1998, Thoma et al. 2018 ^62-65^, Zheng et al. 2021 | Reports on edentulous ridge without implants (5) |
| Baltagioglu et al. 2015 ^66^ | Peri-implantitis (1) |
| Schmitt et al 2021, De Angelis et al. 2021 | Non-randomized study (2) |

**Characteristics of the included research and qualitative results**:

As per the inclusion criteria, all references described an RCT, with the majority of them having been conducted at the University level and a few^67-73^ being conducted in private practice settings. Most of the clinical trials were parallel-designed, with 19 being a split-mouth trial, and one study having done a combination of both^71-73^. 11 of the studies were conducted in Asia, 29 in Europe, 7 in North America, and 13 in South America. Except for 3 studies^74-76^, all others were a single-center RCT, and all provided information on the current primary outcome of soft tissue augmentation at teeth sites (i.e. Gingival thickness (GT)). The included treatment arms were CTG, hADM, pADM, CMX, and VCMX. The range of follow-up for the included treatment arms was from 6 to 144 months (6-month follow-up data from studies which had at least a 12-month recall were also included). It should be noted that due to a lack of unequal distribution of the time points and unequal data after 72 months, data beyond this timepoint was not considered in the analysis.

The characteristics of the included studies on implants is presented in Table 2. Among the included RCTs, most were conducted in Europe, and a few in North America^56, 77-80^, Asia^81^, South America^82^, and Africa^83-86^. Similar to the studies on natural dentition, most were conducted at a university level, and only 4 in a private practice^77, 79, 87^, all but two studies were multi-center^77, 88^, and the majority were university-sponsored. The investigated treatment arms were CTG, hADM, pADM, CMX, VCMX, as well as no augmentation (which served as the negative control). The range of follow-up for the included treatment arms was from 6 to 48 months.

**Quality assessment and “Risk of bias” report**

**Risk of bias in randomized controlled clinical trials around teeth**

The risk of bias assessment across was analyzed separately as per the analysis on teeth, and implants.

Among the included studies for natural dentition, thirty-five were classified as having an overall low risk of bias. While 18 studies raised some concerns. The majority of the studies showed a low risk in domain 5 (reporting of results). Four studies exhibited some concerns in terms of risk of bias in domain 2 (deviations from intended intervention). Domain 3 (D3), which assesses the management of missing outcome data, generally showed low risk across studies, indicating robust handling of missing data.

**Risk of bias in randomized controlled clinical trials around implants**

Among the included studies investigating dental implants, 20 were classified as having an overall low risk of bias, while only 3 presented with some concerns, and 1 showed a high level of bias. Among the 3 studies with some concerns, the pattern was consistent relative to the rating of the domains among two trials which showed a potential risk in all 5 domains, and one which showed concerns in domains 1 (due to randomization). One showed a high risk of bias relative to domain 2. It should be noted that none of the studies were excluded from the analysis solely based on the risk of bias assessment.

**Discussion – Limitations**

Although the present review offers valuable insights into the effectiveness of different soft tissue grafting approaches, certain limitations are inherently tied to the available literature. One major limitation is the lack of consistent and quantifiable data on patient-reported outcome measures (PROMs), including post-operative pain, satisfaction, and healing experiences. While these aspects are critical for clinical decision-making and are increasingly emphasized in contemporary periodontal and implant therapy, heterogeneity in assessment tools and reporting methods precluded meaningful meta-analyses in this area. Standardized reporting of PROMs in future trials will be essential to enable direct comparisons between different graft materials and surgical approaches, ultimately supporting more patient-centered treatment planning. Similarly, it would be beneficial to evaluate clinicians’ subjective assessments of both the clinical utility and handling of these materials, as well as their perception of how such products contribute to clinical care and influence patient experiences. Additionally, few studies reported data beyond soft tissue thickness and keratinized tissue gain—such as esthetic outcomes—thereby limiting the breadth of outcome measures available for comprehensive analysis. Despite these limitations, the findings of the present review provide robust and convincing evidence on the clinical efficacy of soft tissue grafts in enhancing tissue phenotype around teeth and implants.

REFERENCES

1.	Berglundh T, Armitage G, Araujo MG, et al. Peri-implant diseases and conditions: Consensus report of workgroup 4 of the 2017 World Workshop on the Classification of Periodontal and Peri-Implant Diseases and Conditions. *J Clin Periodontol* 2018;45 Suppl 20:S286-S291.

2.	Sterne JAC, Savović J, Page MJ, et al. RoB 2: a revised tool for assessing risk of bias in randomised trials. *Bmj* 2019;366:l4898.

3.	Brożek J, Akl EA, Alonso‐Coello P, et al. Grading quality of evidence and strength of recommendations in clinical practice guidelines: part 1 of 3. An overview of the GRADE approach and grading quality of evidence about interventions. *Allergy* 2009;64:669-677.

4.	Orwin RG. Evaluating coding decisions. In: *The handbook of research synthesis*. New York, NY, US: Russell Sage Foundation, 1994:139-162.

5.	Berlucchi I, Francetti L, Del Fabbro M, Basso M, Weinstein RL. The influence of anatomical features on the outcome of gingival recessions treated with coronally advanced flap and enamel matrix derivative: a 1-year prospective study. *Journal of periodontology* 2005;76:899‐907.

6.	Cordioli G, Mortarino C, Chierico A, Grusovin MG, Majzoub Z. Comparison of 2 techniques of subepithelial connective tissue graft in the treatment of gingival recessions. *Journal of periodontology* 2001;72:1470-1476.

7.	Francetti L, Weinstein R, Taschieri S, Corbella S. Coronally Advanced Flap With or Without Subepithelial Connective Tissue Graft for the Treatment of Single Recession: 5-Year Outcomes from a Comparative Study. *International journal of periodontics & restorative dentistry* 2018;38:819–825.

8.	Hirsch A, Goldstein M, Goultschin J, Boyan BD, Schwartz Z. A 2-year follow-up of root coverage using sub-pedicle acellular dermal matrix allografts and subepithelial connective tissue autografts. *Journal of periodontology* 2005;76:1323‐1328.

9.	Moses O, Artzi Z, Sculean A, Tal H, Kozlovsky A, Romanos GE. Comparative study of two root coverage procedures: a 24-month follow-up multicenter study. *Journal of periodontology* 2006;77:195‐202.

10.	Nemcovsky CE, Artzi Z, Tal H, Kozlovsky A, Moses O. A multicenter comparative study of two root coverage procedures: coronally advanced flap with addition of enamel matrix proteins and subpedicle connective tissue graft. *Journal of periodontology* 2004;75:600‐607.

11.	Pini Prato G, Clauser C, Cortellini P, Tinti C, Vincenzi G, Pagliaro U. Guided tissue regeneration versus mucogingival surgery in the treatment of human buccal recessions. A 4-year follow-up study. *Journal of periodontology* 1996;67:1216-1223.

12.	Pini Prato G, Tinti C, Vincenzi G, Magnani C, Cortellini P, Clauser C. Guided tissue regeneration versus mucogingival surgery in the treatment of human buccal gingival recession. *Journal of periodontology* 1992;63.

13.	Pini-Prato GP, Cairo F, Nieri M, Franceschi D, Rotundo R, Cortellini P. Coronally advanced flap versus connective tissue graft in the treatment of multiple gingival recessions: a split-mouth study with a 5-year follow-up. *Journal of clinical periodontology* 2010;37:644‐650.

14.	Ricci G, Silvestri M, Tinti C, Rasperini G. A clinical/statistical comparison between the subpedicle connective tissue graft method and the guided tissue regeneration technique in root coverage. *The International journal of periodontics & restorative dentistry* 1996;16:538-545.

15.	Carney CM, Rossmann JA, Kerns DG, et al. A comparative study of root defect coverage using an acellular dermal matrix with and without a recombinant human platelet-derived growth factor. *Journal of periodontology* 2012;83:893-901.

16.	Gilbert LR, Lohra P, Mandlik VB, Rath SK, Jha AK. Comparative evaluation of surgical modalities for coverage of gingival recession: an armed forces Medical College perspective. *Armed forces medical journal, india* 2015;71:53‐59.

17.	Gupta A, Kediege S, Mittal A, Jain K, Gupta S, Chaudhry S. Amnion and chorion membranes in the treatment of gingival recession and their effect on gingival biotype: a clinical and ultrasonographic study. *Journal of clinical and diagnostic research* 2018;12:ZC26‐ZC32.

18.	Harris RJ. A comparative study of root coverage obtained with guided tissue regeneration utilizing a bioabsorbable membrane versus the connective tissue with partial-thickness double pedicle graft. *Journal of periodontology* 1997;68.

19.	Harris RJ. A comparative study of root coverage obtained with an acellular dermal matrix versus a connective tissue graft: results of 107 recession defects in 50 consecutively treated patients. *The International journal of periodontics & restorative dentistry* 2000;20:51-59.

20.	Harris RJ. Clinical evaluation of 3 techniques to augment keratinized tissue without root coverage. *Journal of periodontology* 2001;72:932-938.

21.	Harris RJ, Miller LH, Harris CR, Miller RJ. A comparison of three techniques to obtain root coverage on mandibular incisors. *Journal of periodontology* 2005;76:1758‐1767.

22.	Joly JC, Carvalho AM, da Silva RC, Ciotti DL, Cury PR. Root coverage in isolated gingival recessions using autograft versus allograft: a pilot study. *Journal of periodontology* 2007;78:1017-1022.

23.	Keceli HG, Kamak G, Erdemir EO, Evginer MS, Dolgun A. The Adjunctive Effect of Platelet-Rich Fibrin to Connective Tissue Graft in the Treatment of Buccal Recession Defects: Results of a Randomized, Parallel-Group Controlled Trial. *Journal of periodontology* 2015;86:1221-1230.

24.	Kimble KM, Eber RM, Soehren S, Shyr Y, Wang HL. Treatment of gingival recession using a collagen membrane with or without the use of demineralized freeze-dried bone allograft for space maintenance. *Journal of periodontology* 2004;75:210-220.

25.	Lafzi A, Abolfazli N, Faramarzi M, Eyvazi M, Eskandari A, Salehsaber F. Clinical comparison of coronally-advanced flap plus amniotic membrane or subepithelial connective tissue in the treatment of Miller's class I and II gingival recessions: a split-mouth study. *Journal of dental research, dental clinics, dental prospects* 2016;10:162‐168.

26.	Muller HP, Stahl M, Eger T. Root coverage employing an envelope technique or guided tissue regeneration with a bioabsorbable membrane. *Journal of periodontology* 1999;70:743‐751.

27.	Pini-Prato G, Baldi C, Pagliaro U, et al. Coronally advanced flap procedure for root coverage. Treatment of root surface: root planning versus polishing. *Journal of periodontology* 1999;70:1064-1076.

28.	Tatakis DN, Trombelli L. Gingival recession treatment: guided tissue regeneration with bioabsorbable membrane versus connective tissue graft. *Journal of periodontology* 2000;71:299-307.

29.	Tonetti MS, Cortellini P, Pellegrini G, et al. Xenogenic collagen matrix or autologous connective tissue graft as adjunct to coronally advanced flaps for coverage of multiple adjacent gingival recession: Randomized trial assessing non-inferiority in root coverage and superiority in oral health-related quality of life. *Journal of clinical periodontology* 2018;45:78-88.

30.	Woodyard JG, Greenwell H, Hill M, Drisko C, Iasella JM, Scheetz J. The clinical effect of acellular dermal matrix on gingival thickness and root coverage compared to coronally positioned flap alone. *Journal of periodontology* 2004;75:44-56.

31.	Zucchelli G, Mounssif I, Stefanini M, Mele M, Montebugnoli L, Sforza NM. Hand and ultrasonic instrumentation in combination with root-coverage surgery: a comparative controlled randomized clinical trial. *Journal of periodontology* 2009;80:577-585.

32.	Aydinyurt HS, Tekin Y, Ertugrul AS. The effect of enamel matrix derivatives on root coverage: a 12-month follow-up of a randomized clinical trial. *Brazilian oral research* 2019;33:e006.

33.	Costa PP, Alves LB, Souza SL, Grisi MF, Palioto DB, Taba M. Root coverage in smokers with acellular dermal matrix graft and enamel matrix derivative: a 12-month randomized clinical trial. *International journal of periodontics & restorative dentistry* 2016;36:525‐532.

34.	Reino DM, Novaes Jr AB, Maia LP, et al. Treatment of gingival recessions in heavy smokers using two surgical techniques: a controlled clinical trial. *Brazilian dental journal* 2012;23:59‐67.

35.	Baltacıoğlu E, Korkmaz YT, Korkmaz FM, Bağış N. A combined peri-implant plastic surgery approach for implants in the esthetic zone using a novel technique: A series of 10 cases. *Quintessence international (Berlin, Germany : 1985)* 2016;47:307-318.

36.	Migliorati M, Amorfini L, Signori A, Biavati AS, Benedicenti S. Clinical and Aesthetic Outcome with Post-Extractive Implants with or without Soft Tissue Augmentation: A 2-Year Randomized Clinical Trial. *Clinical implant dentistry and related research* 2015;17:983-995.

37.	Park SH, Lee KW, Oh TJ, Misch CE, Shotwell J, Wang HL. Effect of absorbable membranes on sandwich bone augmentation. *Clinical oral implants research* 2008;19:32-41.

38.	Park SH, Wang HL. Clinical significance of incision location on guided bone regeneration: human study. *Journal of periodontology* 2007;78:47-51.

39.	Sanz-Martin I, Encalada C, Sanz-Sanchez I, Aracil J, Sanz M. Soft tissue augmentation at immediate implants using a novel xenogeneic collagen matrix in conjunction with immediate provisional restorations: A prospective case series. *Clinical implant dentistry and related research* 2019;21:145-153.

40.	van Nimwegen WG, Raghoebar GM, Zuiderveld EG, Jung RE, Meijer HJA, Muhlemann S. Immediate placement and provisionalization of implants in the aesthetic zone with or without a connective tissue graft: A 1-year randomized controlled trial and volumetric study. *Clinical oral implants research* 2018;29:671-678.

41.	Yoshino S, Kan JY, Rungcharassaeng K, Roe P, Lozada JL. Effects of connective tissue grafting on the facial gingival level following single immediate implant placement and provisionalization in the esthetic zone: a 1-year randomized controlled prospective study. *The International journal of oral & maxillofacial implants* 2014;29:432-440.

42.	Zuiderveld EG, Meijer HJA, den Hartog L, Vissink A, Raghoebar GM. Effect of connective tissue grafting on peri-implant tissue in single immediate implant sites: A RCT. *Journal of clinical periodontology* 2018;45:253-264.

43.	Almeida ALPF, Esper LA, Sbrana MC, Ribeiro IWJ, Kaizer ROF. Utilization of low-intensity laser during healing of free gingival grafts. *Photomedicine and Laser Surgery* 2009;27:561-564.

44.	Boynuegri D, Nemli SK, Kasko YA. Significance of keratinized mucosa around dental implants: a prospective comparative study. *Clin Oral Implants Res* 2013;24:928-933.

45.	Cornelini R, Barone A, Covani U. Connective tissue grafts in postextraction implants with immediate restoration: a prospective controlled clinical study. *Practical procedures & aesthetic dentistry* 2008;20:337‐343.

46.	Hanser T, Khoury F. Alveolar Ridge Contouring with Free Connective Tissue Graft at Implant Placement: A 5-Year Consecutive Clinical Study. *The International journal of periodontics & restorative dentistry* 2016;36:465-473.

47.	Roccuzzo M, Dalmasso P, Pittoni D, Roccuzzo A. Treatment of buccal soft tissue dehiscence around single implant: 5-year results from a prospective study. *Clinical oral investigations* 2019;23:1977-1983.

48.	Roccuzzo M, Gaudioso L, Bunino M, Dalmasso P. Surgical treatment of buccal soft tissue recessions around single implants: 1-year results from a prospective pilot study. *Clinical oral implants research* 2014;25:641-646.

49.	Schneider D, Grunder U, Ender A, Hämmerle CHF, Jung RE. Volume gain and stability of peri-implant tissue following bone and soft tissue augmentation: 1-year results from a prospective cohort study. *Clinical oral implants research* 2011;22:28-37.

50.	Oh SL, Masri RM, Williams DA, Ji C, Romberg E. Free gingival grafts for implants exhibiting lack of keratinized mucosa: a prospective controlled randomized clinical study. *Journal of clinical periodontology* 2017;44:195-203.

51.	Basegmez C, Ersanli S, Demirel K, Bölükbasi N, Yalcin S. The comparison of two techniques to increase the amount of peri-implant attached mucosa: free gingival grafts versus vestibuloplasty. One-year results from a randomised controlled trial. *European journal of oral implantology* 2012;5:139-145.

52.	Vellis J, Kutkut A, Al-Sabbagh M. Comparison of Xenogeneic Collagen Matrix vs. Free Gingival Grafts to Increase the Zone of Keratinized Mucosa Around Functioning Implants. *Implant dentistry* 2019;28:20-27.

53.	Buyukozdemir Askin S, Berker E, Akincibay H, et al. Necessity of keratinized tissues for dental implants: a clinical, immunological, and radiographic study. *Clinical implant dentistry and related research* 2015;17:1-12.

54.	Lorenzo R, Garcia V, Orsini M, Martin C, Sanz M. Clinical efficacy of a xenogeneic collagen matrix in augmenting keratinized mucosa around implants: a randomized controlled prospective clinical trial. *Clin Oral Implants Res* 2012;23:316-324.

55.	Basegmez C, Karabuda ZC, Demirel K, Yalcin S. The comparison of acellular dermal matrix allografts with free gingival grafts in the augmentation of peri-implant attached mucosa: a randomised controlled trial. *European journal of oral implantology* 2013;6:145-152.

56.	Anderson LE, Inglehart MR, El-Kholy K, Eber R, Wang HL. Implant associated soft tissue defects in the anterior maxilla: a randomized control trial comparing subepithelial connective tissue graft and acellular dermal matrix allograft. *Implant Dent* 2014;23:416-425.

57.	Cairo F, Barbato L, Tonelli P, Batalocco G, Pagavino G, Nieri M. Xenogeneic collagen matrix versus connective tissue graft for buccal soft tissue augmentation at implant site. A randomized, controlled clinical trial. *Journal of clinical periodontology* 2017;44:769-776.

58.	Froum SJ, Khouly I, Tarnow DP, et al. The use of a xenogeneic collagen matrix at the time of implant placement to increase the volume of buccal soft tissue. *The International journal of periodontics & restorative dentistry* 2015;35:179-189.

59.	Puzio M, Błaszczyszyn A, Hadzik J, Dominiak M. Ultrasound assessment of soft tissue augmentation around implants in the aesthetic zone using a connective tissue graft and xenogeneic collagen matrix – 1-year randomised follow-up. *Annals of Anatomy* 2018;217:129-141.

60.	Ustaoglu G, Paksoy T, Gumus KC. Titanium-Prepared Platelet-Rich Fibrin Versus Connective Tissue Graft on Peri-Implant Soft Tissue Thickening and Keratinized Mucosa Width: A Randomized, Controlled Trial. *J Oral Maxillofac Surg* 2020.

61.	Hutton CG, Johnson GK, Barwacz CA, Allareddy V, Avila-Ortiz G. Comparison of two different surgical approaches to increase peri-implant mucosal thickness: A randomized controlled clinical trial. *J Periodontol* 2018;89:807-814.

62.	Akcali A, Schneider D, Unlu F, Bicakci N, Kose T, Hammerle CHF. Soft tissue augmentation of ridge defects in the maxillary anterior area using two different methods: a randomized controlled clinical trial. *Clin Oral Implan Res* 2015;26:688-695.

63.	Artiushkevich AS, Krishtopenko LS. The comparative characteristics of vestibuloplasty methods. *Stomatologiia* 1990;69:54-56.

64.	Barone R, Clauser C, Grassi R, Merli M, Prato GP. A protocol for maintaining or increasing the width of masticatory mucosa around submerged implants: a 1-year prospective study on 53 patients. *The International journal of periodontics & restorative dentistry* 1998;18:377-387.

65.	Thoma DS, Alshihri A, Fontolliet A, Hämmerle CHF, Jung RE, Benic GI. Clinical and histologic evaluation of different approaches to gain keratinized tissue prior to implant placement in fully edentulous patients. *Clinical oral investigations* 2018;22:2111-2119.

66.	Baltacıoğlu E, Bağış B, Korkmaz FM, Aydın G, Yuva P, Korkmaz YT. Peri-Implant Plastic Surgical Approaches to Increasing Keratinized Mucosa Width. *The Journal of oral implantology* 2015;41:e73-e81.

67.	Cardaropoli D, Tamagnone L, Roffredo A, Gaveglio L. Treatment of gingival recession defects using coronally advanced flap with a porcine collagen matrix compared to coronally advanced flap with connective tissue graft: a randomized controlled clinical trial. *J Periodontol* 2012;83:321-328.

68.	Cardaropoli D, Tamagnone L, Roffredo A, Gaveglio L. Coronally advanced flap with and without a xenogenic collagen matrix in the treatment of multiple recessions: a randomized controlled clinical study. *Int J Periodontics Restorative Dent* 2014;34 Suppl 3:s97-102.

69.	Rebele SF, Zuhr O, Schneider D, Jung RE, Hurzeler MB. Tunnel technique with connective tissue graft versus coronally advanced flap with enamel matrix derivative for root coverage: a RCT using 3D digital measuring methods. Part II. Volumetric studies on healing dynamics and gingival dimensions. *J Clin Periodontol* 2014;41:593-603.

70.	Rotundo R, Genzano L, Patel D, D'Aiuto F, Nieri M. Adjunctive benefit of a xenogenic collagen matrix associated with coronally advanced flap for the treatment of multiple gingival recessions: A superiority, assessor-blind, randomized clinical trial. *J Clin Periodontol* 2019;46:1013-1023.

71.	Zuhr O, Akakpo D, Eickholz P, et al. Tunnel technique with connective tissue graft versus coronally advanced flap with enamel matrix derivate for root coverage: 5-year results of an RCT using 3D digital measurement technology for volumetric comparison of soft tissue changes. *J Clin Periodontol* 2021;48:949-961.

72.	Zuhr O, Rebele SF, Schneider D, Jung RE, Hurzeler MB. Tunnel technique with connective tissue graft versus coronally advanced flap with enamel matrix derivative for root coverage: a RCT using 3D digital measuring methods. Part I. Clinical and patient-centred outcomes. *J Clin Periodontol* 2014;41:582-592.

73.	Zuhr O, Rebele SF, Vach K, et al. Tunnel technique with connective tissue graft versus coronally advanced flap with enamel matrix derivate for root coverage: 2-year results of an RCT using 3D digital measuring for volumetric comparison of gingival dimensions. *J Clin Periodontol* 2020;47:1144-1158.

74.	Jepsen K, Stefanini M, Sanz M, Zucchelli G, Jepsen S. Long-Term Stability of Root Coverage by Coronally Advanced Flap Procedures. *J Periodontol* 2017;88:626-633.

75.	Stefanini M, Jepsen K, de Sanctis M, et al. Patient-reported outcomes and aesthetic evaluation of root coverage procedures: a 12-month follow-up of a randomized controlled clinical trial. *J Clin Periodontol* 2016;43:1132-1141.

76.	Wang HL, Romanos GE, Geurs NC, Sullivan A, Suarez-Lopez Del Amo F, Eber RM. Comparison of two differently processed acellular dermal matrix products for root coverage procedures: a prospective, randomized multicenter study. *J Periodontol* 2014;85:1693-1701.

77.	Clem DS, McClain PK, McGuire MK, et al. Harvest graft substitute for soft tissue volume augmentation around existing implants: A randomized, controlled and blinded multicenter trial. *J Periodontol* 2024;95:740-748.

78.	Lee CT, Tran D, Tsukiboshi Y, et al. Clinical efficacy of soft-tissue augmentation on tissue preservation at immediate implant sites: A randomized controlled trial. *J Clin Periodontol* 2023;50:1010-1020.

79.	Oh SL, Ji C, Azad S. Free gingival grafts for implants exhibiting a lack of keratinized mucosa: Extended follow-up of a randomized controlled trial. *J Clin Periodontol* 2020;47:777-785.

80.	Tavelli L, Majzoub J, Kauffmann F, et al. Coronally advanced flap versus tunnel technique for the treatment of peri-implant soft tissue dehiscences with the connective tissue graft: A randomized, controlled clinical trial. *J Clin Periodontol* 2023;50:980-995.

81.	Kassis J, Aboud ME. The Effect of Connective Tissue Graft Compared to Concentrated Growth Factor Graft on Buccal Peri-Implant Gingival Thickness: A 12-month Randomized Controlled Clinical Trial. *J Oral Maxillofac Surg* 2024;82:563-571.

82.	Lazzari TR, Jardini MAN, Castro Dos Santos N, et al. Single implant placement in the maxillary aesthetic area with or without connective tissue grafting: A 1-year follow-up randomised clinical trial. *Int J Oral Implantol (Berl)* 2022;15:57-67.

83.	Abdelwahab D, Ezz AlArab A, El Nahass H. Soft tissue esthetics around immediately provisionalized delayed implants with and without connective tissue graft: A randomized clinical trial pilot study. *Clin Implant Dent Relat Res* 2023;25:11-22.

84.	Hamdy A, Ibrahim SSA, Ghalwash D, Adel-Khattab D. Volumetric assessment of volume stable collagen matrix in maxillary single implant site development: A randomized controlled clinical trial. *Clin Implant Dent Relat Res* 2024;26:930-941.

85.	Happe A, Debring L, Schmidt A, Fehmer V, Neugebauer J. Immediate Implant Placement in Conjunction with Acellular Dermal Matrix or Connective Tissue Graft: A Randomized Controlled Clinical Volumetric Study. *Int J Periodontics Restorative Dent* 2022;42:381-390.

86.	Panwar M, Kosala M, Malik D, Sharma D. Comparison of acellular dermal matrix allografts and connective tissue autografts in soft-tissue augmentation around immediate implants: A pilot study. *Med J Armed Forces India* 2022;78:S251-S257.

87.	Wiesner G, Esposito M, Worthington H, Schlee M. Connective tissue grafts for thickening peri-implant tissues at implant placement. One-year results from an explanatory split-mouth randomised controlled clinical trial. *Eur J Oral Implantol* 2010;3:27-35.

88.	Cosyn J, Eeckhout C, De Bruyckere T, et al. A multi-centre randomized controlled trial comparing connective tissue graft with collagen matrix to increase soft tissue thickness at the buccal aspect of single implants: 1-year results. *J Clin Periodontol* 2022;49:911-921.
